# Supplementary material for: Autophagy lipidation machinery regulates axonal microtubule dynamics but is dispensable for survival of mammalian neurons
Source: Nat Commun. 2020 Mar 24;11:1535. doi: 10.1038/s41467-020-15287-9 (PMC7093409; doi:10.1038/s41467-020-15287-9)
Supplement: Supplementary file 1 — Supplementary Material [file 41467_2020_15287_MOESM1_ESM.pdf]

Autophagy lipidation machinery regulates axonal microtubule dynamics but is dispensable for survival of mammalian neurons

Negrete-Hurtado et al.

Supplementary Table 1. List of antibodies used in the current study

REAGENT or RESOURCE				SOURCE	IDENTIFIER
Antibodies					
	WB	ICC	IHC		
Rabbit polyclonal anti-ATG16L1	1:1000	-	-	MBL	Cat# PM040
Rabbit monoclonal anti-ATG5	1:1000	-	-	abcam	Cat# ab108327
Rabbit polyclonal anti-ATG9	-	1:300	-	Novus Biologicals	Cat# NB110-56893
Rabbit polyclonal anti-ATG13	-	1:300	-	Arigo (Biomol GmbH)	Cat# ARG55112.050
Guinea pig polyclonal anti-Bassoon		1:500	1:500	Synaptic Systems	Cat# 141 004
Rabbit polyclonal anti-CASPASE-3 cleaved (Asp175)	1:1000	-	1:200	Cell Signaling	Cat# 9661
Rabbit polyclonal anti-c-FOS	-	-	1:500	Synaptic Systems	Cat# 226 003
Rabbit polyclonal anti-CLASP2	1:1000	1:500	1:500	Millipore	Cat# ABT263
Goat polyclonal anti-DNCT1	-	1:200	-	abcam	Cat# ab11806
Rabbit polyclonal anti-ELKS1	1:1000	1:500	-	Novus Biologicals	Cat# NBP1-88178
Rabbit monoclonal anti-FIP200	1:1000	1:200	-	Cell Signaling	Cat# 12436
Mouse monoclonal anti-GAPDH	1:1000	-	-	Sigma-Aldrich	Cat# G8795
Chicken polyclonal anti-GFP	-	1:2000	1:1000	abcam	Cat# ab13970
Mouse monoclonal anti-GFP	1:5000	-	-	Takara Bio Clontech	Cat# 632375
Rabbit polyclonal anti-LC3B	1:2000	-	-	Novus Biologicals	Cat# NB600-1384SS
Mouse monoclonal anti-LC3A/B	-	1:300	-	MBL	Cat# M152-3
Mouse monoclonal anti-MAP2	-	1:500	-	Sigma-Aldrich	Cat# M9942
Mouse monoclonal anti-MAPK activated (P-ERK-1&2)	1:1000	-	-	Sigma-Aldrich	Cat# M8159

Rabbit polyclonal anti-mCherry	1:2000	-	-	abcam	Cat# ab167453
Mouse monoclonal anti-mCherry	-	1:5000	-	Novus Biologicals	Cat# NBP1-96752
Rabbit polyclonal anti-P-S6 Ribosomal Protein (Ser235/236)	1:1000	-	-	Cell Signaling	Cat# 2211
Guinea pig polyclonal anti-p62	1:1000	1:500	1:1000	Progen	Cat# GP62-C
Rabbit monoclonal anti-P-AKT (Ser473)	1:1000	-	-	Cell Signaling	Cat# 4060
Rabbit monoclonal anti-RAB7	-	1:250	-	Cell Signaling	Cat#9367S
Rabbit polyclonal anti-SYB2	-	1:800	-	Synaptic Systems	Cat# 104 202
Mouse monoclonal anti-Synapsin 1	-	1:500	-	Synaptic Systems	Cat# 106 001
Rabbit polyclonal anti-P-TRKB (Y816)	1:1000	1:250	-	abcam	Cat# ab75173
Mouse monoclonal anti-α-Tubulin	1:1000	1:500	-	Synaptic Systems	Cat# 302 211
Mouse monoclonal anti- beta-3-Tubulin	-	-	1:500	Thermo Fisher Sci	Cat# 14-4510-80
Rabbit monoclonal anti-α-Tubulin acetylated (Lys40)	1:1000	-	-	Cell Signaling	Cat# 5335
Mouse monoclonal anti-α-Tubulin tyrosinated	1:1000	-	-	Sigma-Aldrich	Cat# T9028
Rabbit polyclonal anti-α-Tubulin detyrosinated	1:1000	-	-	Millipore	Cat# AB3201
Rabbit polyclonal anti-α-Tubulin Δ2	1:1000	1:500	-	Synaptic Systems	Cat# 302 213
Rabbit antiserum anti-Ubiquitin	1:1000	1:200	-	Sigma-Aldrich	Cat# U5379
Mouse monoclonal anti-WIPI2	-	1:300	-	BioRad	Cat# AB_10845951
Goat anti-Mouse IgG (H+L) peroxidase-conjugated	1:5000	-	-	Jackson ImmunoResearch	Cat# 115-035-003

Goat anti-Mouse IgG, light chain specific, peroxidase conjugated	1:5000	-	-	Jackson ImmunoResearch	Cat# 115-035-174
Goat anti-Rabbit IgG (H+L) peroxidase-conjugated	1:5000	-	-	Jackson ImmunoResearch	Cat# 111-035-003
Goat anti-Guinea Pig IgG (H+L) peroxidase-conjugated	1:5000	-	-	Jackson ImmunoResearch	Cat# 106-035-003
Normal Rabbit IgG	1:5000	-	-	Cell Signaling	Cat# 2729
Normal Mouse IgG	1:5000	-	-	Millipore	Cat# 12-371
Alexa Fluor 488 Goat anti-Mouse IgG	-	1:500	-	Thermo Fisher Sci	Cat# A-11029
Alexa Fluor 488 Goat anti-Rabbit IgG	-	1:500	1:500	Thermo Fisher Sci	Cat# A-11034
Alexa Fluor 488 Goat anti-Chicken IgG	-	1:500	1:500	Thermo Fisher Sci	Cat# A-11039
Alexa Fluor 568 Goat anti-Mouse IgG	-	1:500	1:500	Thermo Fisher Sci	Cat# A-11031
Alexa Fluor 568 Goat anti-Rabbit IgG	-	1:500	1:500	Thermo Fisher Sci	Cat# A-11036
Alexa Fluor 568 Donkey anti-Goat IgG	-	1:500	1:500	Thermo Fisher Sci	Cat# A-11057
Alexa Fluor 647 Goat anti-Mouse IgG	-	1:500	1:500	Thermo Fisher Sci	Cat# A-21236
Alexa Fluor 647 Goat anti-Rabbit IgG	-	1:500	1:500	Thermo Fisher Sci	Cat# A-21245
Alexa Fluor 647 Goat anti-Guinea Pig IgG	-	1:500	1:500	Thermo Fisher Sci	Cat# A-21450
Alexa Fluor 647 Goat anti-Rat IgG		1:500		Thermo Fisher Sci	Cat# A-21247
Abberior STAR 635P Goat anti-mouse IgG	-	1:1000	-	Abberior	Cat#ST635P

Supplementary Table 2. List of oligonucleotides, vectors and primers used in the current study

AAV9-*CamKIIα*-eGFP	Penn Vector Core. University of Pennsylvania School of Medicine	AV-9-pV1917
Oligonucleotides		
siRNA *Clasp2* smart pool	Dharmacon	L-062336-01-0005
siRNA *Erc1* (*Elks1*) smart pool	Dharmacon	L-058829-01-0005
siRNA *Maplc3b* smart pool	Dharmacon	M-040989-01-0005
siRNA *Mallc3a* smart pool	Dharmacon	L-056203-01-0005
siRNA *Fip200* smart pool	Dharmacon	L-041191-01-0005
siRNA Scramble non-targeting smart pool	Dharmacon	D-001206-13-05
Primer: *Bdnf* Forward: GGGTCACAGCGGCAGATAAA	This paper	N/A
Primer: *Bdnf* Reverse: GCCTTTGGATACCGGGACTT	This paper	N/A
Primer: *Ntrk2* Forward: CCGCTAGGATTTGGTGTACTG	PrimerBank	ID: 6679150a1
Primer: *Ntrk2* Reverse: CCGGGTCAACGCTGTTAGG	PrimerBank	ID: 6679150a1
Primer: *Gapdh* Forward: AACTTTGGCATTGTGGAAGG	[1]	N/A
Primer: *Gapdh* Reverse: ACACATTGGGGGTAGGAACA	[1]	N/A
Cre-Primer for genotyping:		
Gen.Cre_1: GAACCTGATGGACATGTTCAGG	eurofins	N/A
Gen.Cre_2: AGTGCGTTCGAACGCTAGAGCCTGT	eurofins	N/A
Gen.Cre_3: TTACGTCCATCGTGGACA	eurofins	N/A
Gen.Cre_4: GGCTGGGTGTTAGCC	eurofins	N/A
Atg5-Primer for genotyping:		
*Atg5*_1: AATATGAAGGCACACCCCTGAAATG	eurofins	N/A
*Atg5*_2: ACAACGTCGAGCACGCTGCGCAAGG	eurofins	N/A
*Atg5*_3: GTACTGCATAATGGTTTAACTCTTGC	eurofins	N/A
Atg16L1-Primer for genotyping:		
*Atg16L1*_1:CAGAATAATTTCCGGCAGAGACCGG	eurofins	N/A

*Atg16L1*_2:AGCCAAAGAAGGAAGGTAAGCA ACGAA	eurofins	N/A
Oligonucleotides and primers for mutagenesis and cloning		
Oligo: *Lc3b G120A* Forward (mutagenesis): CCCAGGAGACGTTCGCGACAGCACTGGCTT	This paper	N/A
Oligo: *Lc3b G120A* Reverse (mutagenesis): ACAGCCAGTGCTGTCGCGAACGTCTCCTGG	This paper	N/A
Oligo: *Lc3b G120A*-stop Forward (mutagenesis): AGGAGACGTTCGCGTAAGCACTGGCTGTTC	This paper	N/A
Oligo: *Lc3b G120A*-stop Reverse (mutagenesis): GTAACAGCCAGTGCTTACGCGAACGTCTCT	This paper	N/A
Primer: *Lc3a* in pEGFP2 Forward (cloning): CGGAATTCATGCCCTCAGACCGGCC	This paper	N/A
Primer: *Lc3a* in pEGFP2 Reverse (cloning): GCGGATCCTCAGAAGCCGAAGGTTTC	This paper	N/A
Oligo: *Lc3a G120A* Forward (mutagenesis): CCCAGGAAACCTTCGCCTTCTGAGGATCCC	This paper	N/A
Oligo: *Lc3a G120A* Reverse (mutagenesis): GTGGATCCTCAGAAGGCGAAGGTTTCCTGG	This paper	N/A
Primer: *Gabarap* in pEGFP2 Forward (cloning): CAAGAAGCTTCATGAAGTTCGTGTACAAA	This paper	N/A
Primer: *Gabarap* in pEGFP2 Reverse (cloning): CTGTGGATCCTCACAGACCATAGACGCTTC	This paper	N/A
Oligo: *Gabarap G116A* Forward (mutagenesis): TGAAAGCGTCTATGCTCTGTGAGGATCCAC	This paper	N/A
Oligo: *Gabarap G116A* Reverse (mutagenesis): GGTGGATCCTCACAGAGCATAGACGCTTTA	This paper	N/A
Recombinant DNA		
Plasmid: pFUGW-H1-eGFP	[2]	N/A
Plasmid: pmCherry-N1	Kind gift from Dr. M. Kreutz	N/A
Plasmid: ptagRFP-C	Kind gift from Dr. M. Kreutz	N/A
Plasmid: pmStrawberry-ATG4BC74A (mouse)	Addgene	#21076
Plasmid: TRKB-mRFP (mouse)	[3]	N/A
Plasmid: EB3-tdTomato (human)	Addgene	#50708

Plasmid: Tubulin-eGFP (chicken)	Addgene	#66105
Plasmid: pEGFP-C1-mApg5 (mouse)	Kind gift from Prof. M. Lammers	N/A
Plasmid: Mito-mCherry	Kind gift from Prof. E. Rugarli	N/A
Plasmid: ptagRFP-LC3B (pro LC3) (rat)	This paper	N/A
Plasmid: ptagRFP-LC3BG120A (rat)	This paper	N/A
Plasmid: eGFP-LC3B (rat)	Kind gift from Dr. M. Kreutz	N/A
Plasmid: eGFP-C-LC3BG120A (rat)	This paper	N/A
Plasmid: eGFP-LC3A (human)	This paper	N/A
Plasmid: eGFP-LC3AG120A (human)	This paper	N/A
Plasmid: eGFP-GABARAP (mouse)	This paper	N/A
Plasmid: eGFP-GABARAP-G116A (mouse)	This paper	N/A
Plasmid: tdTomato-ELKS1 (rat)	Kind gift from Dr. H. Kawabe and Prof. Nils Brose	N/A
Plasmid: SYB2-pHluorin (rat)	[2]	N/A

Supplementary Table 3. Total number of cells in N experiments

Main Figures					
2e	WT: 40<br>KO: 40	2h	WT: 234<br>KO: 238	2j	Control: 486<br>$ATG4B^{C74A}$: 360
2k	Scr siRNA: 534<br>*Fip200* siRNA: 820	2q	LC3 WT: 332	2s	Scr siRNA: 417

			LC3 G120: 251		*Lc3b* siRNA: 502
3e (ATG5)	WT: 27<br>KO: 33	3e (ATG16L1)	WT: 31<br>KO: 28	3h (ATG5)	WT: 29<br>KO: 30
3h (ATG16L1)	WT: 31<br>KO: 22	3k	WT: 27<br>KO: 25	3m	WT: 28<br>KO: 27
3s and 3r	29 per condition				
4c	WT^{eGFP}: 25<br>KO^{eGFP}:26<br>WT$^{eGFP\text{-}ATG5}$: 20<br>KO$^{eGFP\text{-}ATG5}$: 24	4p	WT: 27<br>KO: 26	4r	eGFP: 30<br>eGFP-LC3B: 30<br>eGFP-LC3BG120A: 30
4t	eGFP: 19<br>eGFP-LC3BG120A: 17<br>eGFP-GABARAPG116A: 20				
6j	KO^{Scr}: 247<br>KO$^{Lc3b\ siRNA}$: 281	6k	WT^{Scr}: 27<br>KO^{Scr}: 27<br>WT$^{Elks1\ siRNA}$: 26<br>KO$^{Elks1\ siRNA}$: 28	6m (number spheroids)	KO^{Scr}: 142<br>KO$^{Elks1\ siRNA}$: 125
6q	KO^{Scr}: 30<br>KO$^{Elks1\ siRNA}$: 26	6s	eGFP: 35<br>eGFP-CLASP2: 39	6t, 6u, 6v	eGFP: 33<br>eGFP-CLASP2: 29
6h	WT: 65<br>KO: 74				
Supplementary figures					
2e	WT: 40<br>KO: 40	2g	WT: 40<br>KO: 40	2m	Scr peptide: 42<br>LC3B peptide: 46
3d	WT: 30<br>KO: 30	3i, 3m, 3o	WT: 30<br>KO: 30	3k	WT: 30<br>KO: 27
3p (boutons)	WT: 359<br>KO: 358	3r (synapses)	WT: 60<br>KO: 60	3t	WT: 13<br>KO: 13
3u	WT: 20<br>KO: 20	3w	WT: 31<br>KO: 28	3y (% moving)	DMSO: 16<br>Ciliobrevin D: 15

3z (retrograde)	DMSO: 15<br>Ciliobrevin D: 9	3z	DIV6: 24<br>DIV12: 20	3b’	WT: 33<br>KO: 28
6p	WT^{Scr}: 19<br>KO^{Scr}: 19<br>$WT^{\textit{Elks1}\ KD}$: 16<br>$KO^{\textit{Elks1}\ KD}$: 17	6r	KO^{Scr}: 37<br>$KO^{\textit{Lc3a}\ KD}$: 33	6v	Scr: 25<br>CLASP2 KD: 25
7a	WT+BDNF: 40<br>KO+BDNF: 40	7b, 7b, 7d	WT+H2O: 32<br>KO+H2O: 36<br>WT+BDNF:33<br>KO+BDNF: 35		

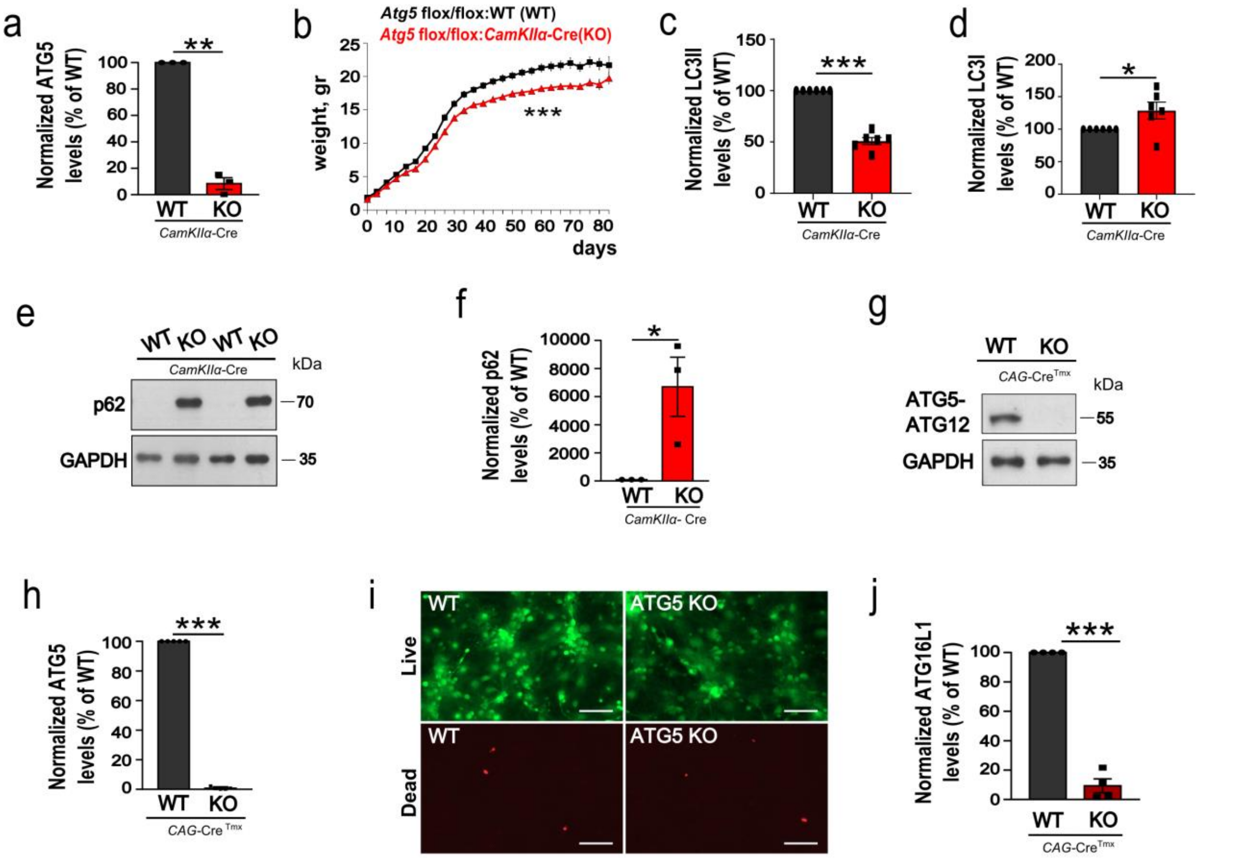

Supplementary Figure 1. Lack of apoptosis in ATG5 and ATG16L1 KO neurons. (**a**) ATG5 expression levels are significantly decreased in cortical brain lysates from 13- week-old ATG5 KO mice compared to controls. Protein levels were quantified relative to GAPDH and the levels in the KO were normalized to the WT set to 100% (KO: 8.40±4.39%). **p=0.001. N=3 independent experiments. (**b**) Growth curves of WT and ATG5KO mice (KO=22, WT=28 animals). ***p<0.000. (**c**) LC3II expression levels are significantly decreased in cortical brain lysates from 13- week-old ATG5 KO mice compared to controls. Protein levels were quantified relative to α-Tubulin and the levels in the KO were normalized to the WT set to 100% (KO: 50.87±3.45%). ***p=0.000. N=6 independent experiments. (**d**) LC3I expression levels are significantly increased in cortical brain lysates from 13- week-old ATG5 KO mice compared to controls. Protein levels

were quantified relative to α-Tubulin and the levels in the KO were normalized to the WT set to 100% (KO: 128.59±13.00%). *p=0.040. N=6 independent experiments. (**e**,**f**) p62 expression levels are significantly increased in cortical brain lysates from 13 week-old ATG5 KO mice compared to controls. Protein levels were quantified relative to GAPDH and the levels in the KO were normalized to the WT set to 100% (KO: 6696.69±2105.47%). *p=0.044. N=3 independent experiments. (**g,h**) ATG5 expression levels are significantly decreased in lysates from cultured ATG5 KO neurons. Protein levels in the KO were quantified relative to GAPDH and the levels in the KO were normalized to the WT set to 100% (KO: 1±0.00%). ***p <0.000. N=5 independent experiments. (**i**) Representative fluorescence images of WT and ATG5 KO primary neurons after performing Live/Dead cell viability assay. Scale bar, 150µm. (**j**) ATG16L1 expression levels are significantly decreased in lysates from cultured ATG16L1 KO neurons. Protein levels in KO condition were normalized to the WT set to 100% (KO:9.34±4.69%). ***p<0.000. N=4 independent experiments.

All graphs show mean ± SEM, statistical analysis was performed by one-sample Student's *t*-test in (a,c,d,f,h,j) and unpaired two-tailed Student's *t*-test in (b). n.s.-non-significant. Source data are provided as a Source Data file.

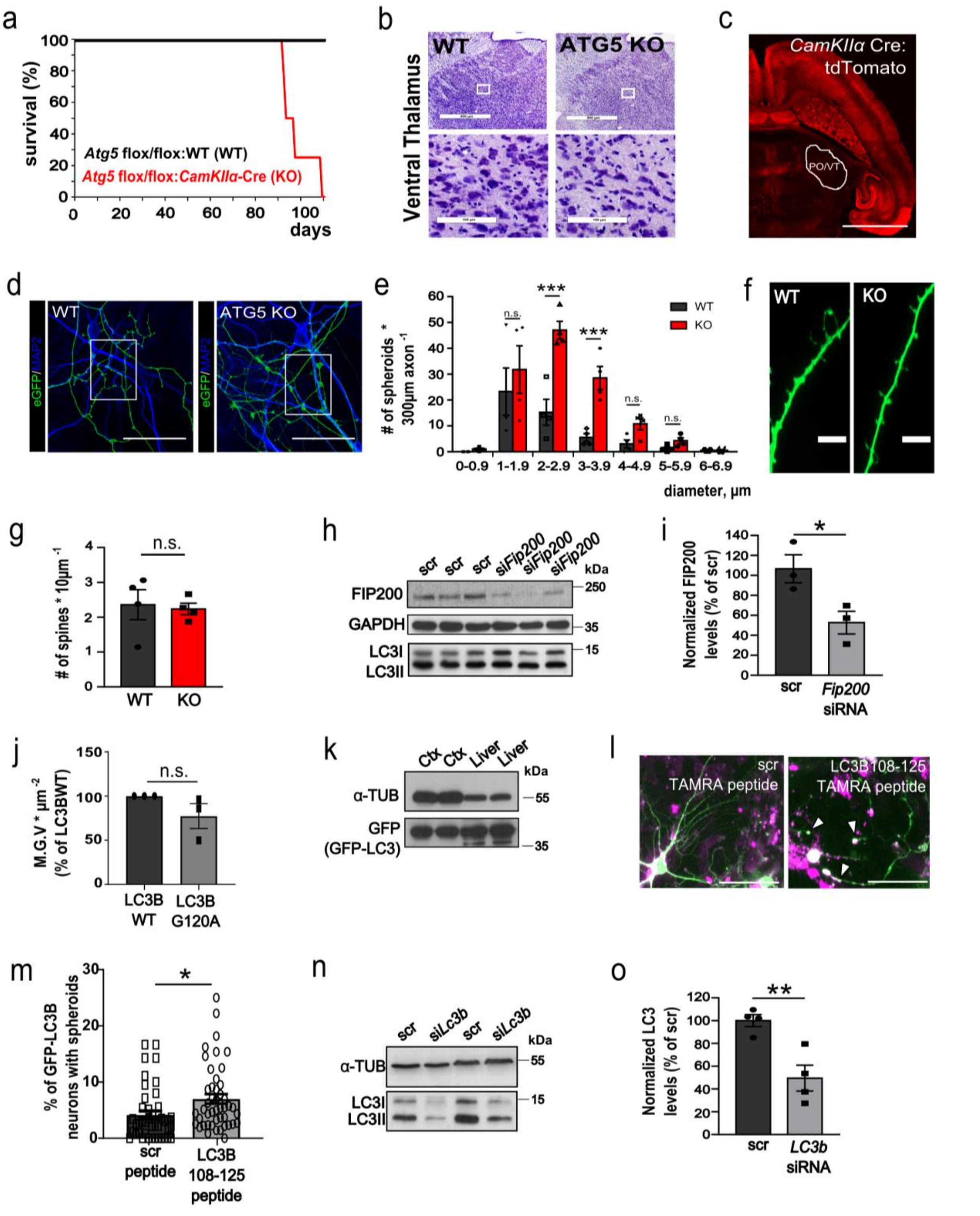

Supplementary Figure 2. Axonal pathology of ATG5 KO cortical neurons. (**a**) Kaplan-Meier survival curves of ATG5 WT and KO mice. (**b**) Loss of ATG5 in forebrain excitatory neurons causes degeneration of thalamic nuclei. Scale bars: 900 µm, inserts 100 µm. (**c**) Cre expression in

the brain of *CamkIIa*:Cre/Rosa-tdTomato mice. Scale bar, 1 mm. (**d**) Primary neurons from WT and ATG5KO mice transfected with eGFP and immunostained for MAP2. Rectangular boxes indicate the areas magnified in Fig. 2d. Scale bars, 50 µm. (**e**) Histogram showing the number of axonal swellings in WT and ATG5KO neurons plotted as a function of their diameter. ***pWT vs KO<0.0000 (for 2-2.99), ***pWT vs KO=0.0009 (for 3-3.99). N=4 independent experiments. (**f,g**) Spine density in WT and ATG5 KO neurons. N=4 independent experiments. Scale bar, 5 µm. (**h,i**) Expression levels of FIP200 in MEF cells transfected either with scr or *Fip200* siRNA. *p=0.042. N=3 independent experiments. (**j**) Expression levels of wild type LC3B and LC3BG120A in cultured neurons. p=0.2489. N=3 independent experiments. (**k**) Expression levels of GFP-LC3 in the cortex (Ctx) and the liver from 10 weeks old GFP-LC3 mice. Samples arise from the same experiment and the blots were processed in parallel, such that one loading control was used. (**l**) Representative images of eGFP-LC3B transgenic neurons treated with LC3B 108-125 or scramble (scr) TAMRA peptides. The red channel intensity was overexposed to illustrate the TAMRA signal within the neurons. Scale bar, 50µm. (**m**) Percentage of eGFP-LC3B neurons with spheroids. *p=0.012. 42-46 images per group (containing in total 1971 (Scr) and 2871 (LC3B) neurons) from N=3 independent experiments. Scale bar, 50µm. (**n,o**) Expression levels of LC3 in MEF cells transfected either with scr (100±4.5%) or *Lc3b* siRNA (49.48±9.86%). **p=0.007. N=4 independent experiments).

All graphs show mean ± SEM, statistical analysis was performed by unpaired two-tailed Student's *t*-test in (g,m), and two-way ANOVA for multiple comparisons in (e) and one-sample Student's *t*-test in (i,j,o). In (i,j,o) KO was normalized to the WT set to 100%. n.s.-non-significant. Total number of neurons in N experiments is shown in Supplementary Table 3. Source data are provided as a Source Data file.

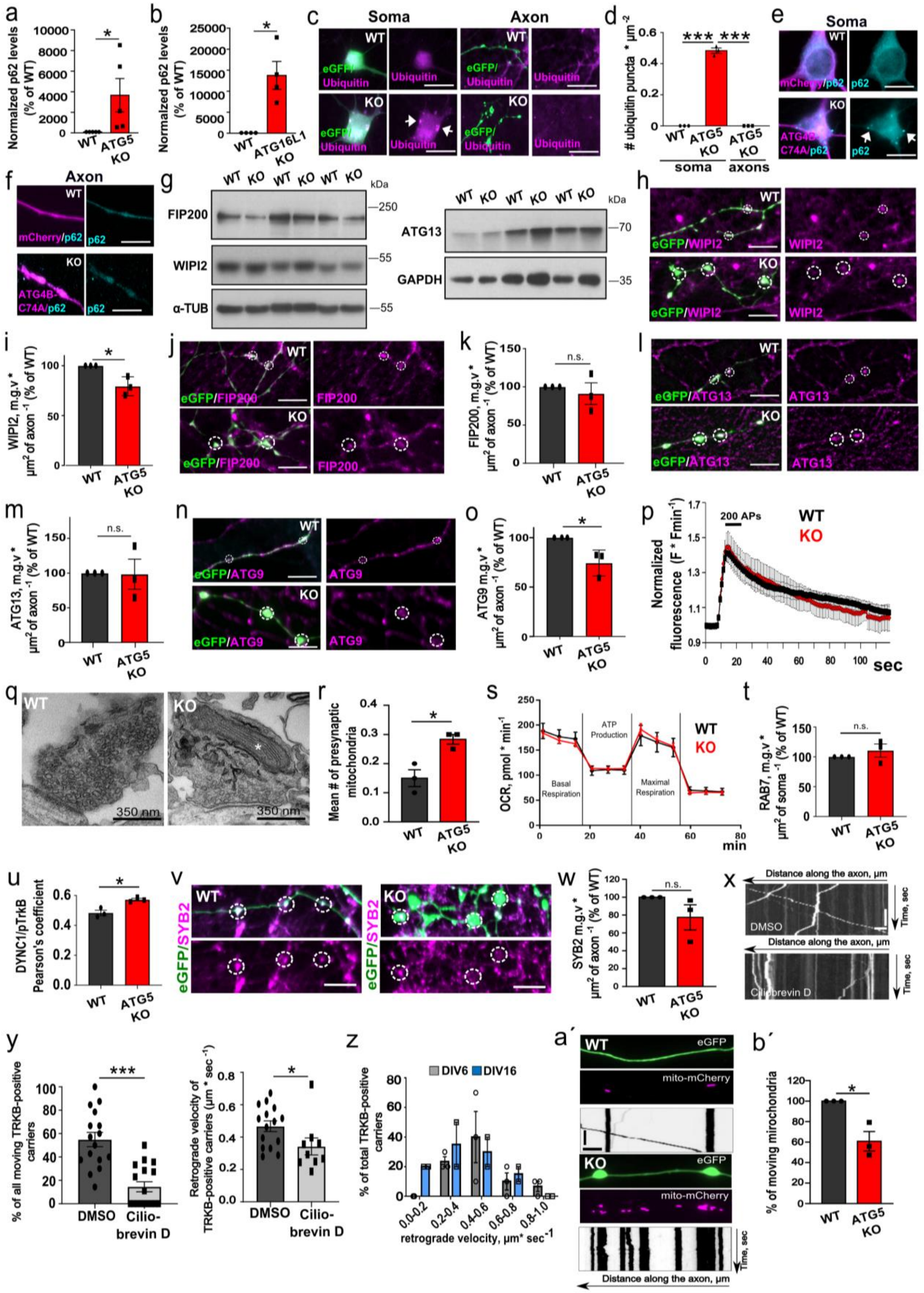

a
Normalzed p62 levels (% of WT)
WT
ATG5 KO
b
Normalzed p62 levels (% of WT)
WT
ATG16L1 KO
c
Soma
Axon
eGFP/Ubiquitin
Ubiquitin
d
ubiquitin puncta * µm-2
soma
axons
e
Soma
mCherry/p62
p62
ATG4B-C74A/p62
f
Axon
mCherry/p62
p62
ATG4B-C74A/p62
g
FIP200
WIPI2
α-TUB
ATG13
GAPDH
kDa
h
eGFP/WIPI2
WIPI2
i
WIPI2, m.g.v * µm2 of axon -1 (% of WT)
j
eGFP/FIP200
FIP200
k
FIP200, m.g.v * µm2 of axon -1 (% of WT)
n.s.
l
eGFP/ATG13
ATG13
m
ATG13, m.g.v * µm2 of axon -1 (% of WT)
n.s.
n
eGFP/ATG9
ATG9
o
ATG9 m.g.v * µm2 of axon -1 (% of WT)
p
200 APs
WT
KO
Normalized fluorescence (F * Fmin-1)
sec
q
WT
KO
350 nm
r
Mean # of presynaptic mitochondria
s
OCR, pmol * min-1
Basal Respiration
ATP Production
Maximal Respiration
min
t
RAB7, m.g.v * µm2 of soma -1 (% of WT)
n.s.
u
DYNC1/pTrkB Pearson's coefficient
v
eGFP/SYB2
w
SYB2 m.g.v * µm2 of axon -1 (% of WT)
n.s.
x
Distance along the axon, µm
Time, sec
DMSO
Ciliobrevin D
y
% of all moving TRKB-positive carriers
Retrograde velocity of TRKB-positive carriers (µm * sec-1)
DMSO
Cilio-brevin D
z
DIV6
DIV16
% of total TRKB-positive carriers
retrograde velocity, µm* sec-1
a´
eGFP
mito-mCherry
Time, sec
Distance along the axon, µm
b´
% of moving mirochondria

Supplementary Figure 3. Functional mitochondria accumulate at ATG5 KO synapses. (**a,b**) p62 protein levels in WT and ATG5 KO (*p=0.046, N=5 independent experiments) or ATG16L1 KO (*p=0.013, N=4 independent experiments) lysates. (**c,d**) Ubiquitin puncta density in ATG5 KO neurons (***p WT^{soma} vs KO^{soma} =0.000; ***p KO^{soma} vs KO^{axon} =0.000). (**e,f**) mCherry- or pmStrawberry-$ATG4B^{C74A}$-expressing neurons, immunostained for p62. (**g**) Protein levels of FIP200, WIPI2 and ATG13 in WT and ATG5 KO lysates. (**h-o**) Immunofluorescent levels of WIPI2 (*p=0.032), FIP200 (p=0.298), ATG13 (p=0.474) and ATG9 (KO: *p=0.039) in eGFP-expressing WT and ATG5 KO axons. (**p**) SYB2-pHluorin responses to 200 APs at 50 Hz in WT and ATG5 KO neurons. (**q,r**) Number of mitochondria within 500nm of the AZ in WT and ATG5 KO neurons (*p=0.016). (**s**) Mitochondrial function in cultured WT and ATG5 KO neurons, measured by Seahorse Assay. 4 wells with 20000 cells per group. (**t**) RAB7 levels in WT or ATG5 KO somata (p=0.219). (**u**) Colocalization of DYNC1 and p816TRKB in WT and ATG5 KO axons, measured by Pearson's correlation coefficient (*p=0.011). (**v,w**) SYB2 levels in WT and ATG5 KO axons (p=0.124). Scale bar, 5µm. (**x**) Time-lapse images of WT axons treated with DMSO or 20µM Ciliobrevin D for 3h. (**y**) Percentage of moving TRKB vesicles (***p<0.0001) and their retrograde velocity in neurons, treated either with DMSO or Ciliobrevin D (*p=0.045). 16 DMSO and 17 CiliobrevinD neurons from N=2 independent experiments. (**z**) Retrograde velocity of TRKB vesicles in DIV6 (24 neurons, N=3, independent experiments) and DIV16 neurons (20 neurons, N=2 independent experiments). (**a´,b´**) Relative axonal mobility of Mito-mCherry in ATG5 KO neurons is significantly decreased compared to controls (*p=0.027). Scale bar, 5µm (snapshots).

Data in (d,i,j,k,m,o,p,r,t,u,w,b`) are from N=3 independent experiments. All graphs show mean ± SEM, statistical analysis was performed by unpaired two-tailed Student's *t*-test in (r,u,y)

and one-sample Student's *t*-test in (a,b,d,I,k,m,o,t,w,b´). In (a,b,d,I,k,m,o,t,w,b´) KO was normalized to the WT set to 100%. Scale bars in (c,e,f,h,j,l,n) 10µm. Total number of neurons in N experiments is shown in Supplementary Table 3. Source data are provided as a Source Data file. n.s.-non-significant. Kymographs scale bar: 5µm x 20sec.

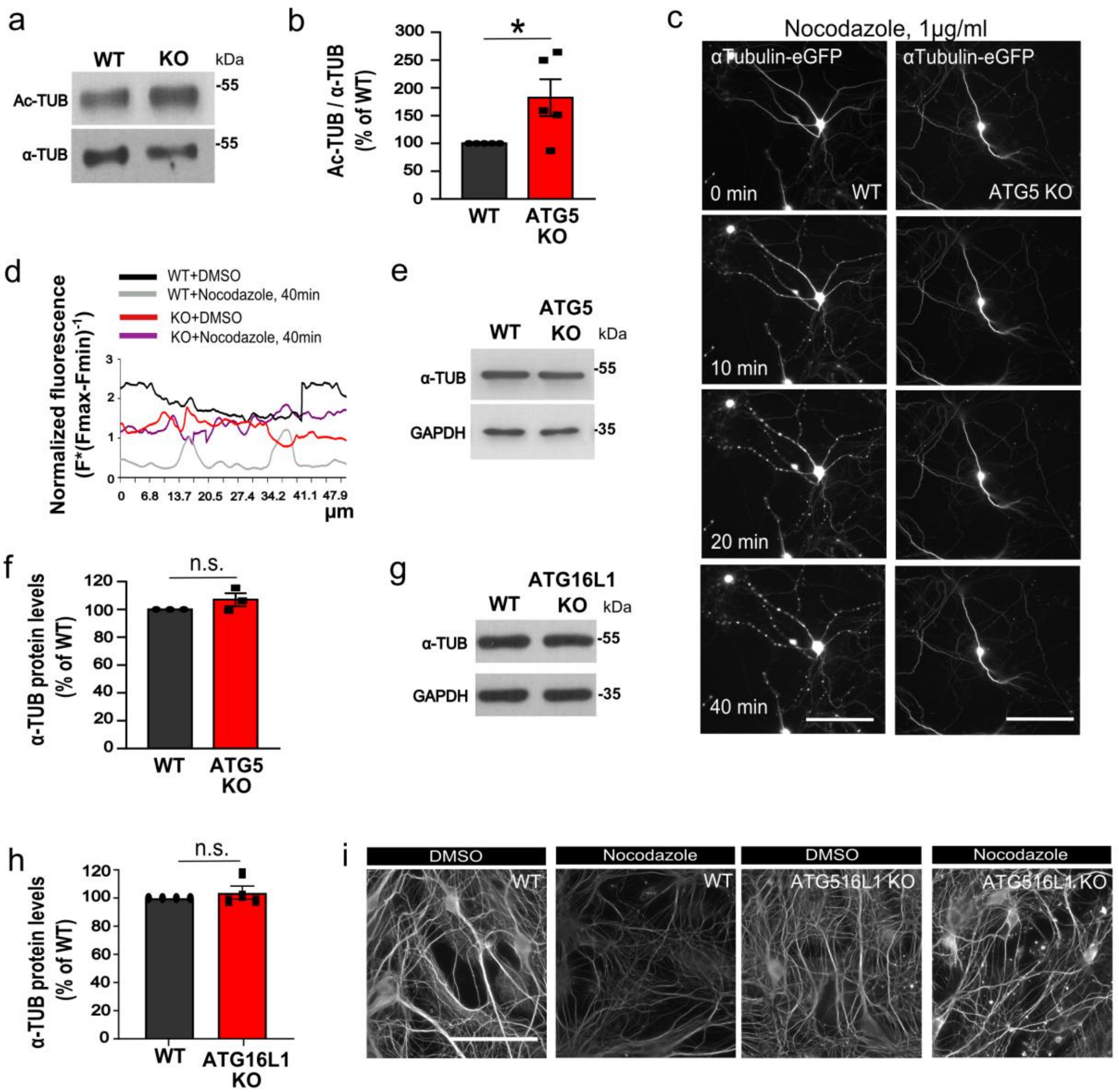

Supplementary Figure 4. Hyperstability of microtubules in ATG5 and ATG16L1 KO neurons. (**a, b**) Levels of acetylated tubulin are significantly increased in lysates from cultured

ATG5 KO neurons compared to controls (KO: 182.25±33.07%). Protein levels in KO condition were normalized to the WT set to 100%. *p=0.034. N=5 independent experiments. (**c**) Time-lapse images of WT and ATG5 KO neurons transfected with αTubulin-eGFP and treated with 1μg/ml of Nocodazole for 40 min. Scale bars, 100μm. (**d**) In WT neurons treated with nocodazole a profound loss of αTubulin-eGFP immunofluorescence along the neurites is detected. In contrast, ATG5 KO neurons treated with Nocodazole were indistinguishable from DMSO-treated controls. (**e,f**) Levels of total α-tubulin are not altered in ATG5 KO neurons compared to controls. Protein levels were normalized to GAPDH and the levels in the KO condition were normalized to the WT set to 100% (KO: 106.99±4.69%). p=0.137. N=4 independent experiments. (**g,h**). Levels of α-tubulin are not altered in ATG16 KO neurons compared to controls. Protein levels were normalized to GAPDH and the levels in the KO condition were normalized to the WT set to 100% (KO: 103.90±4.64%, p=0.436, N=4 independent experiments). (**i**) Immunofluorescence images of WT and ATG16L1KO neurons treated with 0.2μg/ml Nocodazole for 1h or with DMSO as a control and immunostained for α-Tubulin. Scale bar, 50μm.

All graphs show mean ± SEM, statistical analysis was performed one-sample Student's *t*-test. n.s.-non-significant. Source data are provided as a Source Data file.

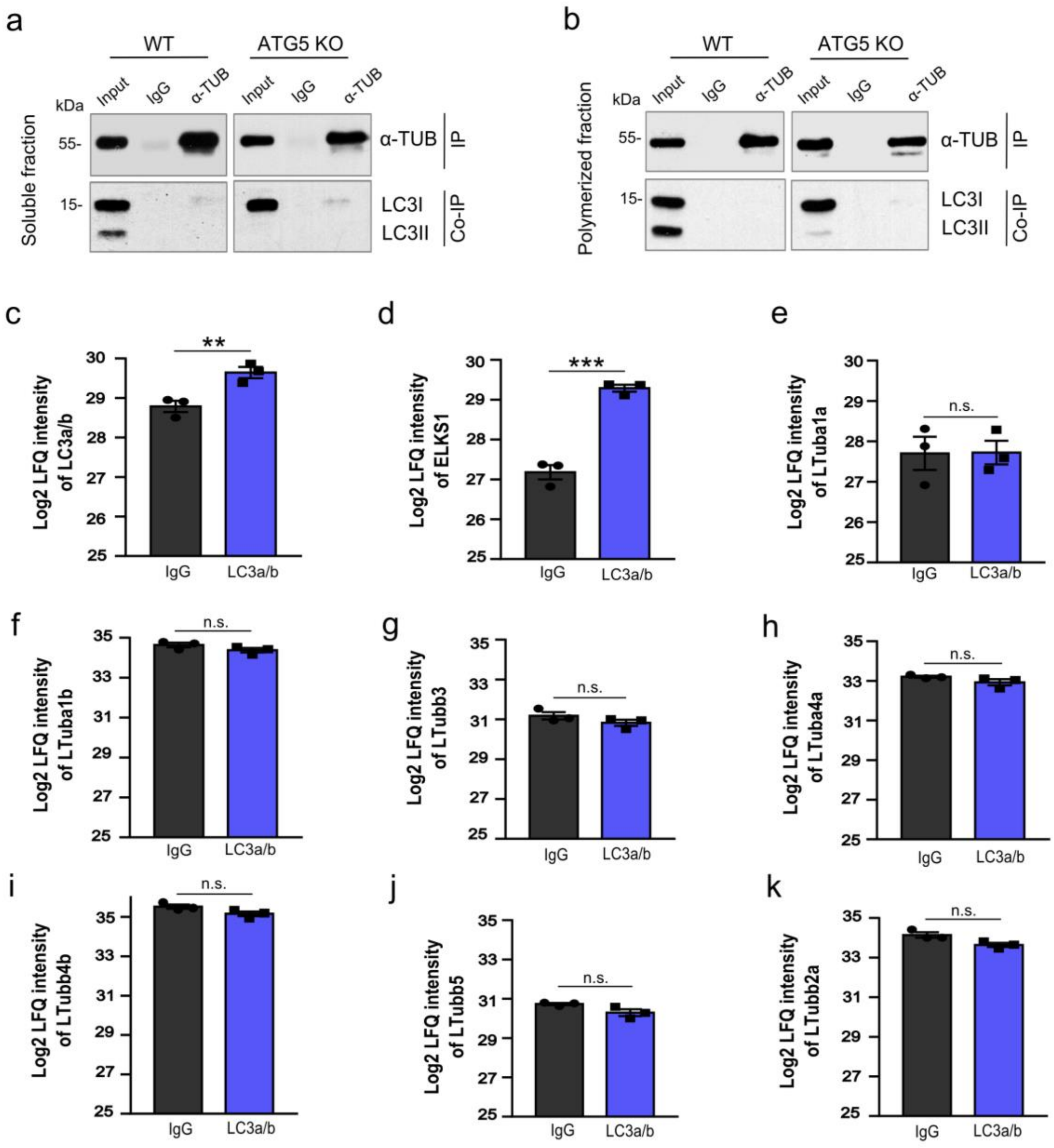

Supplementary Figure 5. Cytoplasmic LC3a/b associates with ELKS1, but not with tubulin in neurons. (**a**) Co-immunoprecipitation of endogenous LC3 with α-Tubulin in soluble microtubules isolated from cultured WT and ATG5 KO neurons. Input, 5% of lysate was added to the assay. Example from N=3 experiments. (**b**) Co-immunoprecipitation of endogenous LC3 with α-Tubulin in polymerized microtubules isolated from cultured WT and ATG5 KO neurons. Input, 8% of lysate was added to the assay. Example from N=3 independent experiments. (**c-k**) Mass

spectrometry analysis of LC3 interaction partners (LC3a/b) or control IgG antibody (IgG) in the brain (N=3 brains for each condition). Log2 LFQ (label-free quantification) intensity denote change of expression of the protein (LC3a/b: **p=0.006; ELKS1: ***p<0.000). N=3 independent experiments.

All graphs show mean ± SEM, statistical analysis was performed by unpaired two-tailed Student's *t*-test. n.s.-non-significant. Source data are provided as a Source Data file.

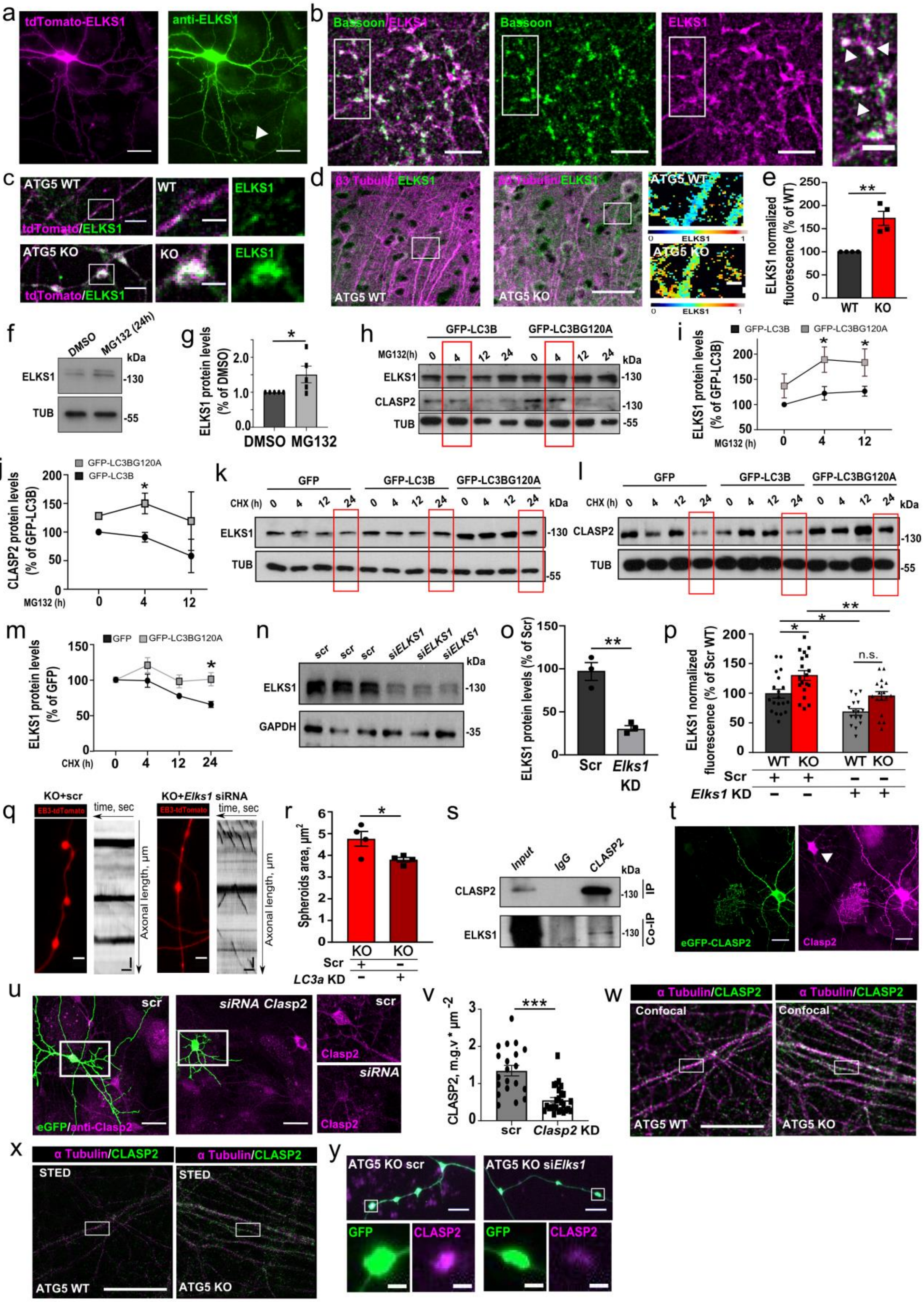
a
tdTomato-ELKS1
anti-ELKS1
b
Bassoon/ELKS1
Bassoon
ELKS1
c
ATG5 WT
tdTomato/ELKS1
WT
ELKS1
ATG5 KO
tdTomato/ELKS1
KO
ELKS1
d
β3 Tubulin/ELKS1
ATG5 WT
ATG5 KO
e
ELKS1 normalized fluorescence (% of WT)
WT
KO
f
DMSO
MG132 (24h)
kDa
ELKS1
TUB
-130
-55
g
ELKS1 protein levels (% of DMSO)
DMSO
MG132
h
GFP-LC3B
GFP-LC3BG120A
MG132(h)
ELKS1
CLASP2
TUB
i
GFP-LC3B
GFP-LC3BG120A
ELKS1 protein levels (% of GFP-LC3B)
MG132 (h)
j
GFP-LC3BG120A
GFP-LC3B
CLASP2 protein levels (% of GFP-LC3B)
MG132 (h)
k
GFP
GFP-LC3B
GFP-LC3BG120A
CHX (h)
ELKS1
TUB
l
GFP
GFP-LC3B
GFP-LC3BG120A
CHX (h)
CLASP2
TUB
m
GFP
GFP-LC3BG120A
ELKS1 protein levels (% of GFP)
CHX (h)
n
scr
siELKS1
ELKS1
GAPDH
o
ELKS1 protein levels (% of Scr)
Scr
Elks1 KD
p
ELKS1 normalized fluorescence (% of Scr WT)
n.s.
WT
KO
Scr
Elks1 KD
q
KO+scr
KO+Elks1 siRNA
EB3-tdTomato
time, sec
Axonal length, µm
r
Spheroids area, µm²
KO
Scr
LC3a KD
s
Input
IgG
CLASP2
CLASP2
ELKS1
IP
Co-IP
t
eGFP-CLASP2
Clasp2
u
scr
siRNA Clasp2
eGFP/anti-Clasp2
Clasp2
siRNA
v
CLASP2, m.g.v * µm⁻²
scr
Clasp2 KD
w
α Tubulin/CLASP2
Confocal
ATG5 WT
ATG5 KO
x
α Tubulin/CLASP2
STED
ATG5 WT
ATG5 KO
y
ATG5 KO scr
ATG5 KO siElks1
GFP
CLASP2

Supplementary figure 6. Cytoplasmic LC3 prevents ELKS1 proteasomal degradation. (a) ELKS1 antibody recognizes overexpressed tdTomato-ELKS1. Arrow indicates untransfected neuron. (**b)** Bassoon and ELKS1 immunostaining in cultured neurons. (**c**) WT and ATG5 KO neurons, immunostained for ELKS1. (**d**) WT and ATG5 KO cortices immunostained for ELKS1 and β3-Tubulin. In the right panel, ELKS1 intensity was false color-coded. (**e**) ELKS1 levels in WT and ATG5 KO brains (**p=0.009). **(f,g)** ELKS1 levels in MG132-treated NSC34 cells (*p=0.049). (**h-j**) ELKS1 and CLASP2 levels in MG132-treated GFP-LC3B- or GFP-LC3B G120A-expressing HEK239T cells. *p^{ELKS1}(4h)=0.012, *p^{ELKS1}(12h)=0.031, *p^{CLASP2}(4h)=0.016. Samples arise from the same experiment and the blots were processed in parallel, such that one loading control was used. (**k-m**) ELKS1 and CLASP2 stability in cyclohexamide-treated GFP-, GFP-LC3B- or GFP-LC3BG120A-expressing HEK 293T cells. *p^{eGFP}(0h) vs ^{eGFP}(24h)=0.013, *p ^{eGFP}(24h) vs $^{eGFP\text{-}LC3BG120A}$(24h)=0.033. (**n,o**) ELKS1 levels in NSC34 cells treated with scr or *Elks1*siRNA. **p=0.004. (**p**) ELKS1 levels in WT or ATG5 KO axons treated with scr (WT^{Scr}, KO^{Scr}, 19 neurons each) or *Elks1* siRNA ($WT^{Elks1\ KD}$, 16 neurons, $KO^{Elks1\ KD}$, 17 neurons). *pWT^{Scr} vs KO^{Scr}= 0.016, pWT^{Scr} vs *$WT^{Elks1\ KD}$=0.023, **p KO^{Scr} vs $KO^{Elks1\ KD}$= 0.007. (**q**) EB3-tdTomato-expressing ATG5 KO neurons treated with scr or *Elks1* siRNA. Scale bars, x: 5µm, y: 15sec. (**r**) Spheroid area in ATG5 KO neurons treated with scr or *Lc3a* siRNA, *p=0.035. (**s**) ELKS1 and CLASP2 co-IP in mouse brain. Input, 4% of lysate. (**t**) CLASP2 antibody recognizes overexpressed eGFP-CLASP2. Arrow indicates untransfected neuron. (**u,v**) CLASP2 is decreased in *Clasp2* siRNA-treated neurons. ***p<0.000. 20 for scr and 25 for siRNA neurons. (**w**, **x**) Confocal and STED images of neurons. (**y**) CLASP2 levels in ATG5 KO axons treated with scr or *Elks*1 siRNA.

Data in (i,j,m,o,v) come from N=3, in (e,r) from N=4, in (g) from N=5, in (p,v) from N=2 independent experiments. Graphs show mean ± SEM, statistical analysis was performed by one-sample Student's *t*-test in (e,g,o,p), two-way ANOVA for multiple comparisons in (i,j,m) and unpaired two-tailed Student's *t*-test in (r,v). Source data are provided as a Source Data file. Scale bars: (a,t) 20µm, (b,y) 5µm, inserts 2µm, (c,w,x) 10µm, inserts 4µm, (d) 100µm, (u) 50µm.

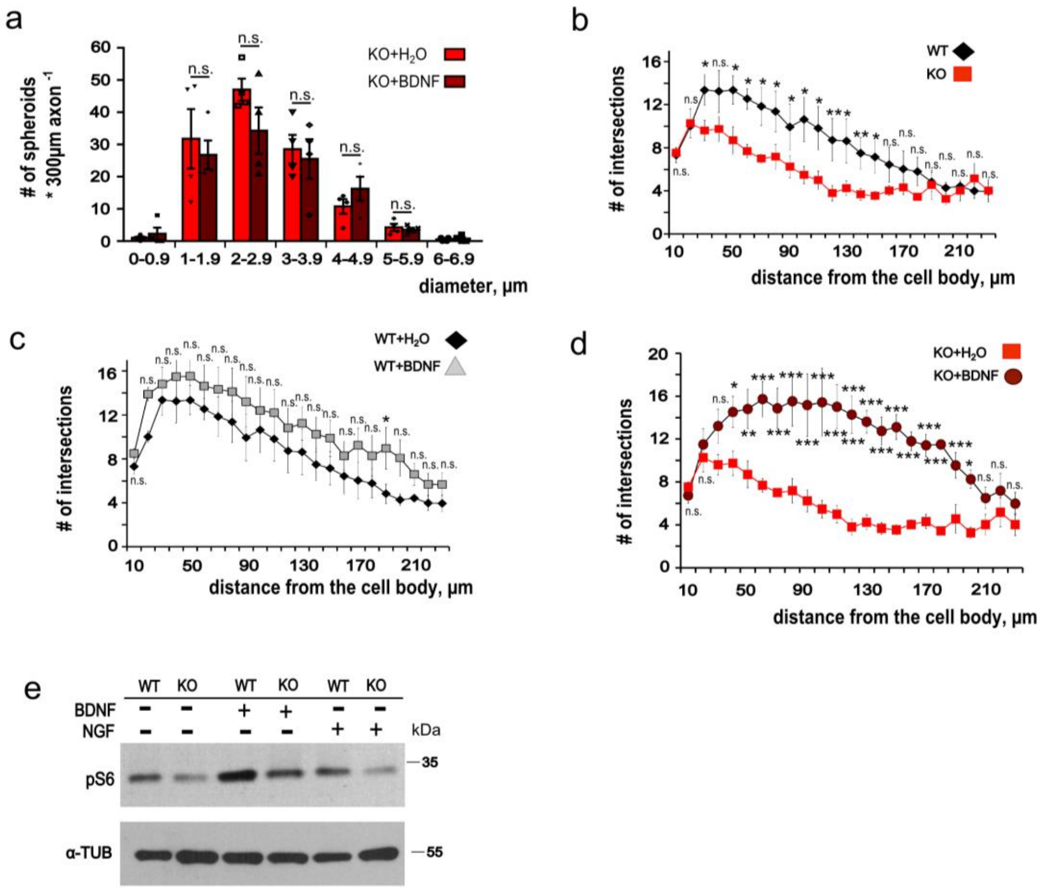

Supplementary figure 7. BDNF rescues defective branching complexity in autophagy-deficient neurons. (**a**) Unaltered number of axonal swellings in ATG5 KO neurons treated with 50ng/ml of BDNF compared to H_2O-treated KO controls. N=4 independent experiments. (**b-d**) Sholl analysis of cultured WT and ATG5 KO neurons at DIV18 reveals the decrease in neuronal

complexity upon loss of ATG5 (b), a phenotype rescued via long-term application of 50 ng/ml of BDNF (**c,d**). N=4 independent experiments. * indicates $p \leq 0.05$, ** indicates $p \leq 0.01$, *** indicates $p \leq 0.001$. (**e**) Immunoblot illustrating the long-term effect of either BDNF (50ng/ml) or NGF (50ng/ml) treatment on the levels of phosphorylated S6 Ribosomal Protein (pS6) in cultured WT and ATG5 KO neurons.

All data shown represent the mean ± SEM. Statistical significance for all data was tested by two-way ANOVA repeated measures for multiple comparisons. Total number of neurons in N experiments is shown in Supplementary Table 3. Source data are provided as a Source Data file. n.s.-non-significant.

Figure 1a

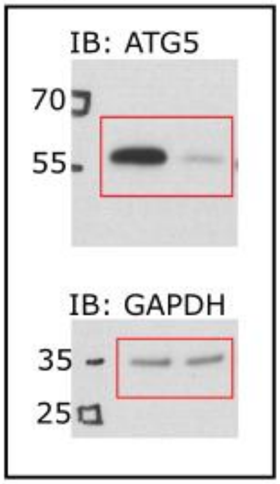

Figure 1b

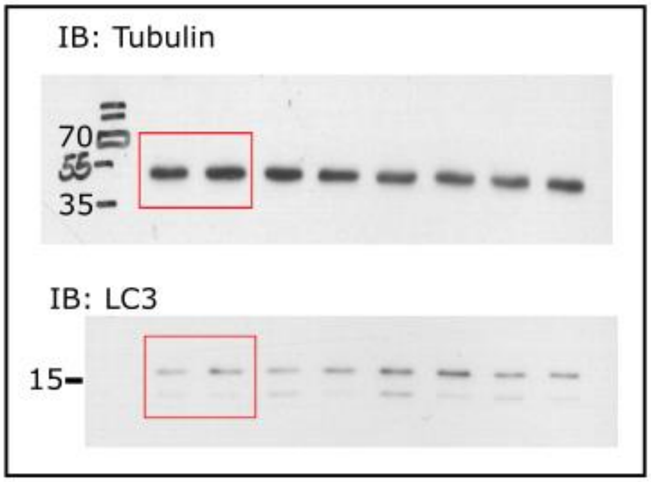

Figure 1h

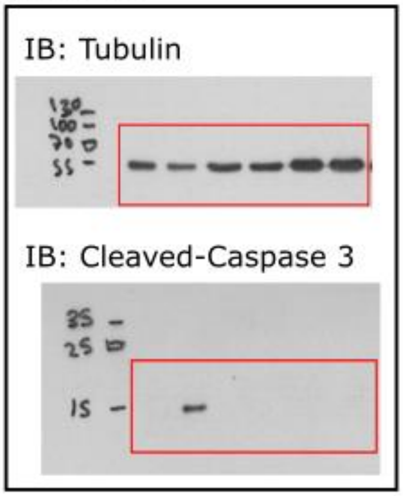

Figure 1k

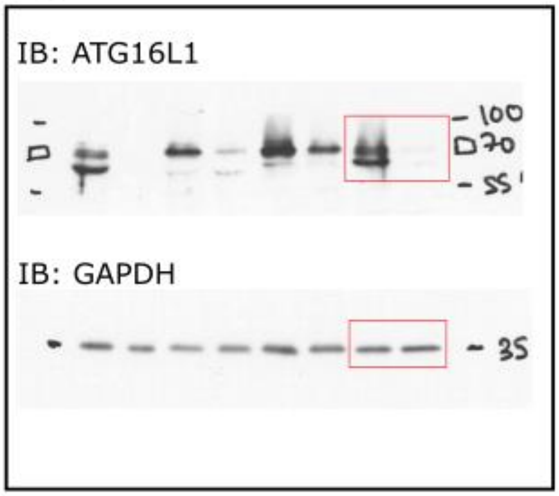

Figure 1l

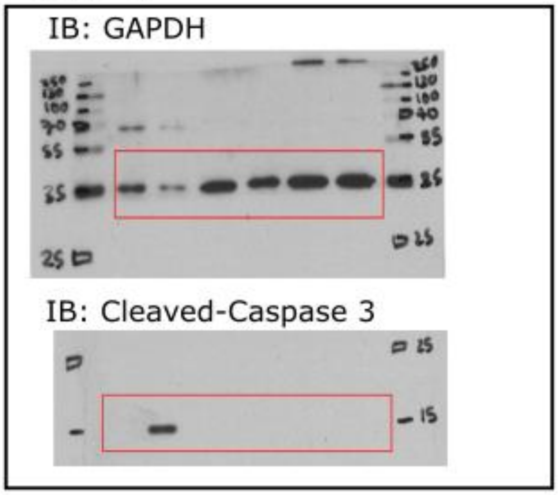

immunoblots from Figure 1

Figure 2l

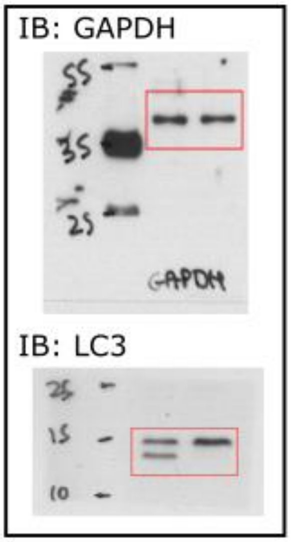

Figure 2n

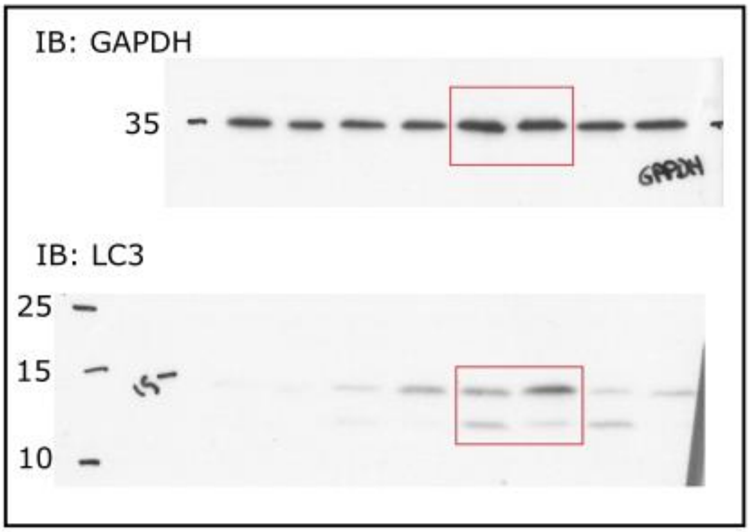

Uncropped immunoblots from Figure 2

Figure 3a

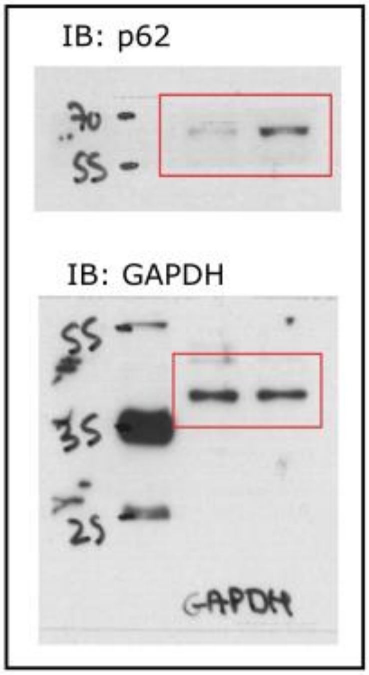

Figure 3b

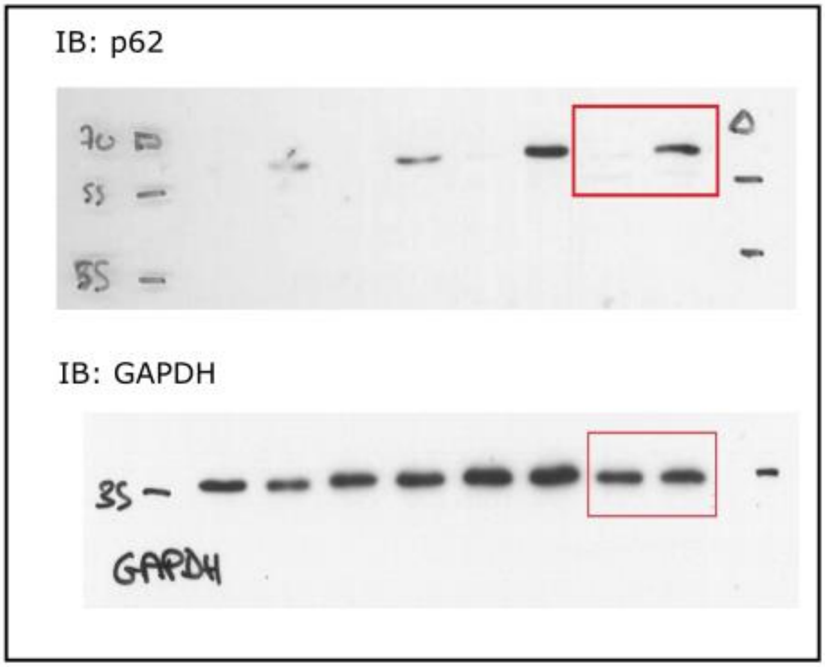

Uncropped immunoblots from Figure 3

Figure 4d

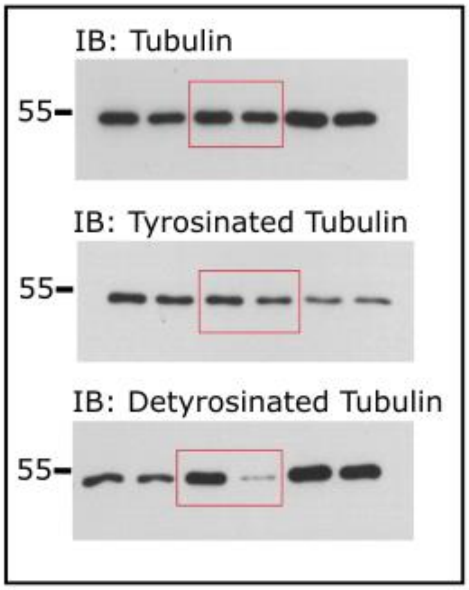

Figure 4g

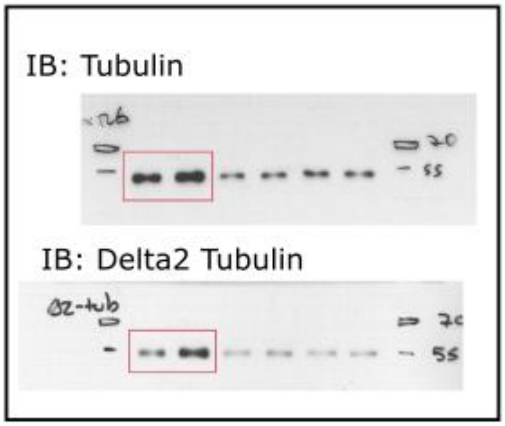

Figure 4i

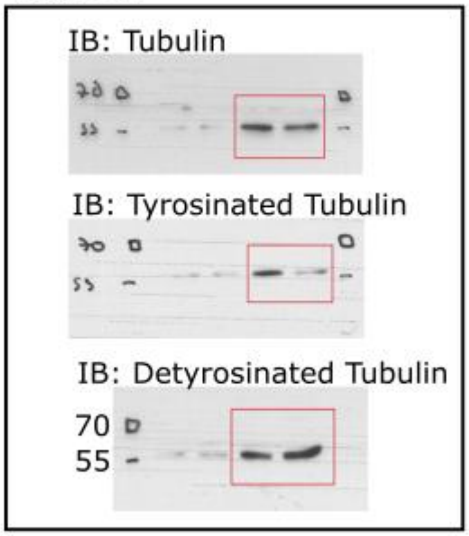

Figure 4l

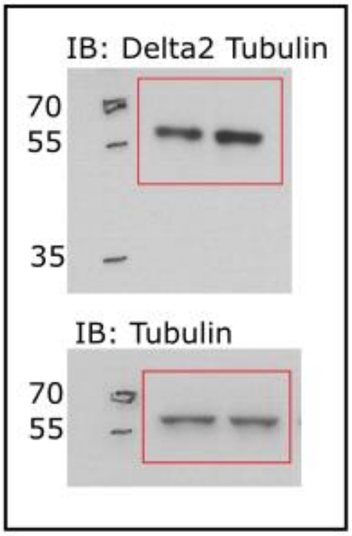

[illegible]

Figure 5b

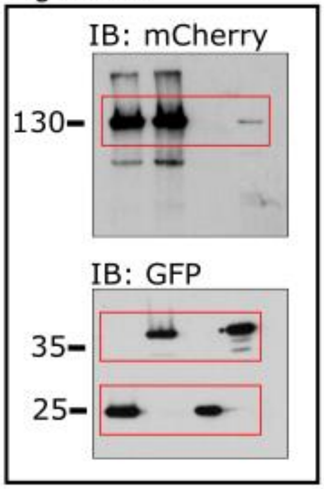

Figure 5c

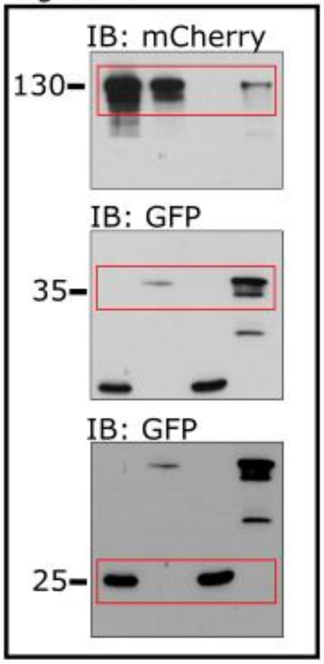

Figure 5d

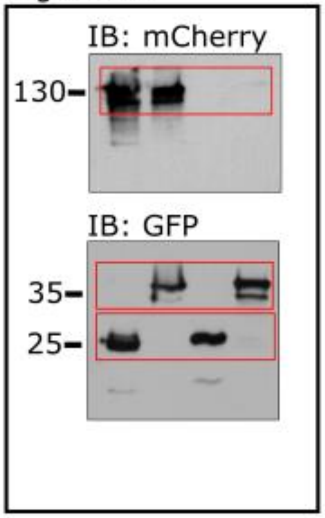

Figure 5e

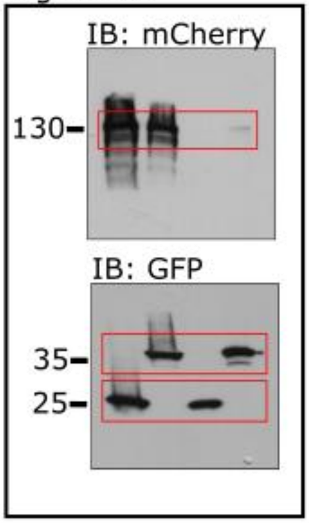

Figure 5f

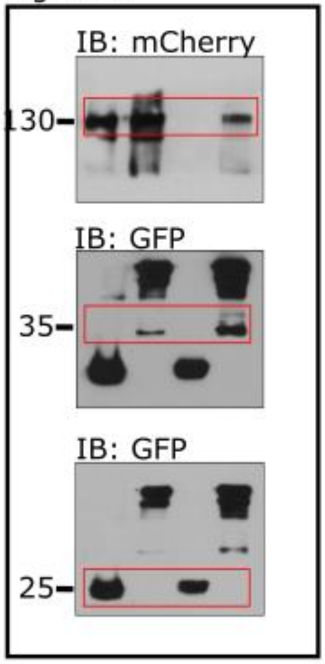

Figure 5g

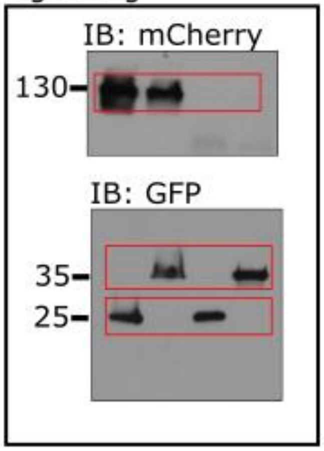

Figure 5h

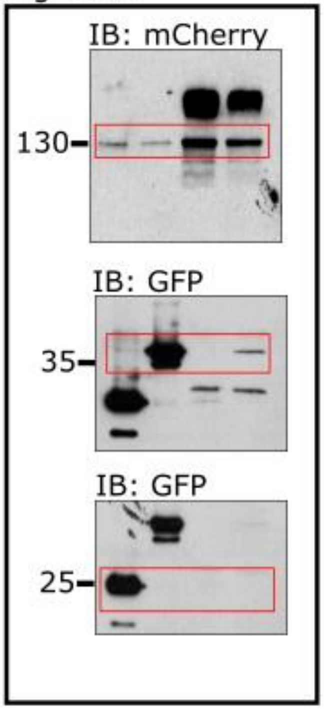

Figure 5i

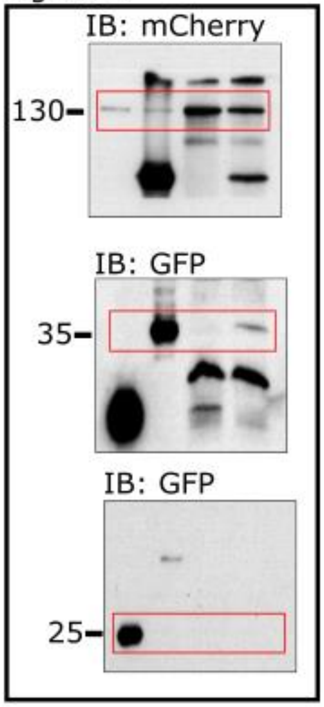

Figure 5j

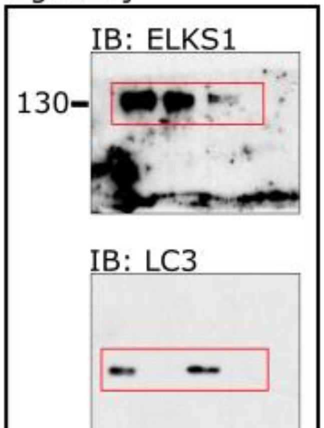

Figure 5k

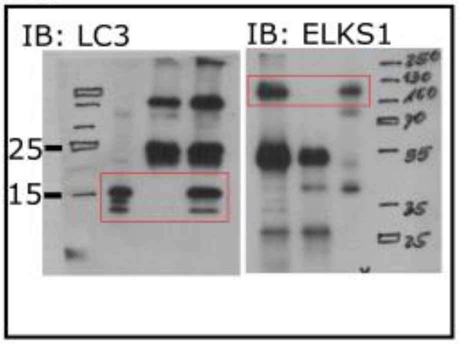

Figure 5l

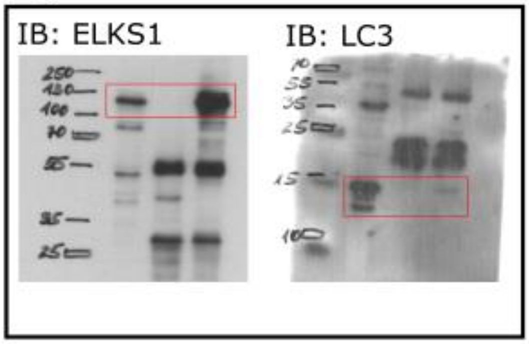

Figure 6a

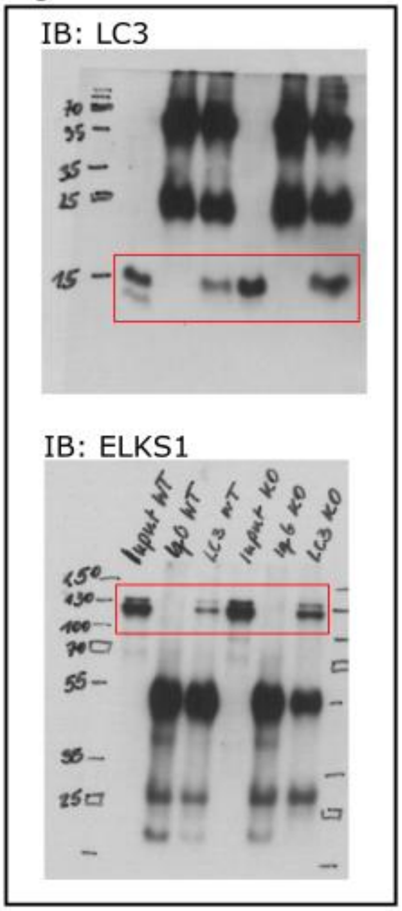

Figure 6b

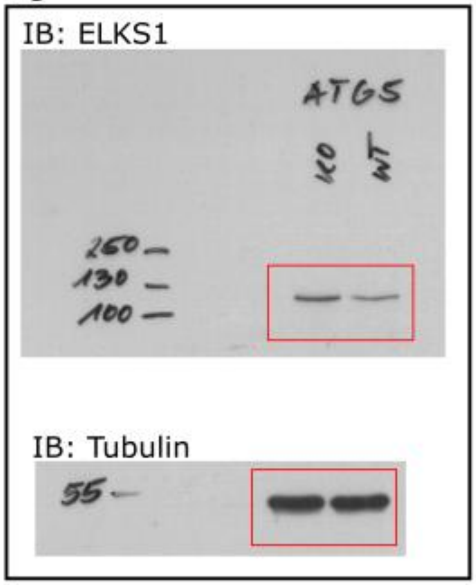

Figure 6g

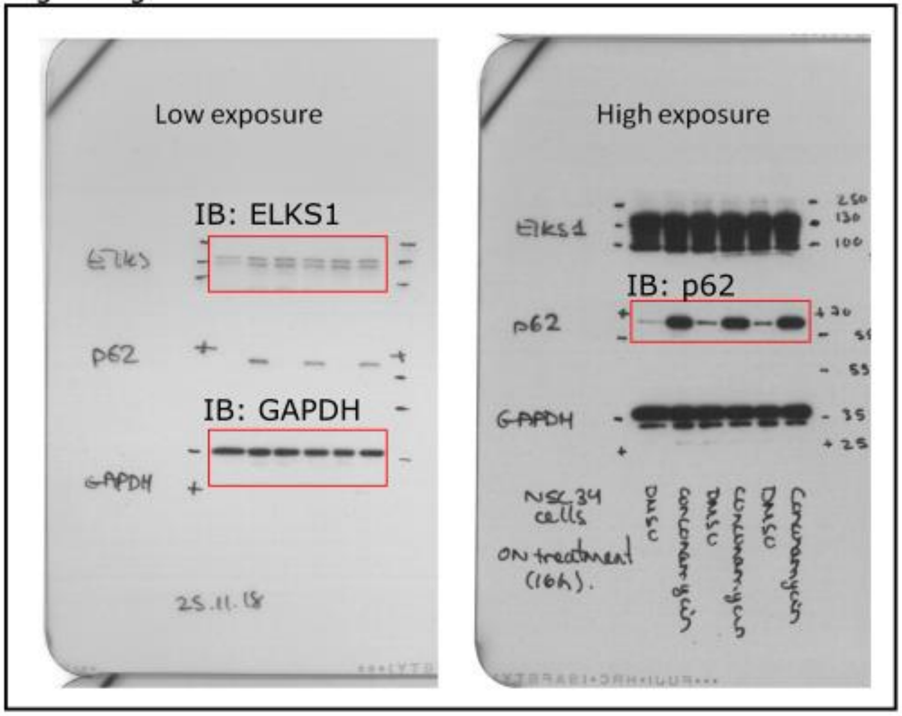

Figure 7a

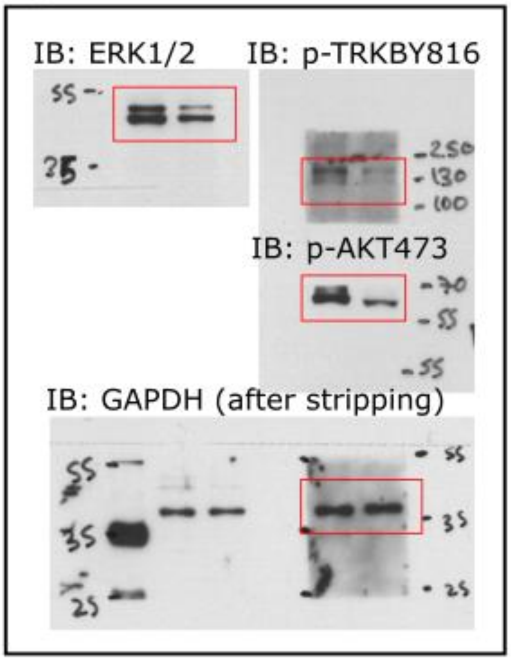

Figure 7e

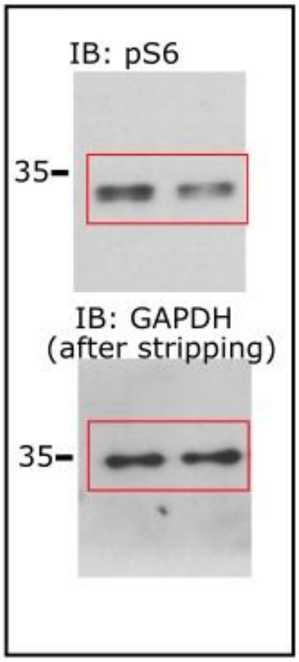

Figure 7i

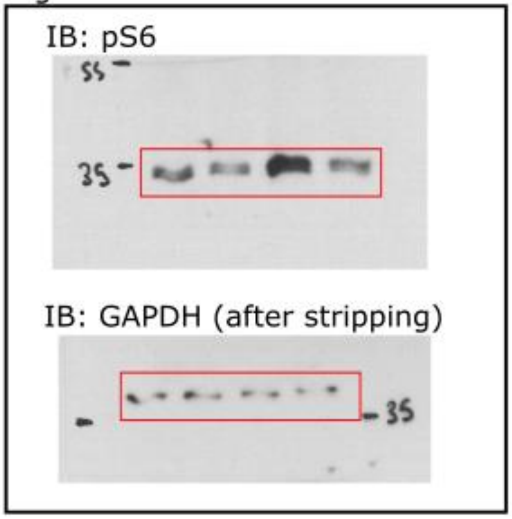

Supplementary Figure 1e

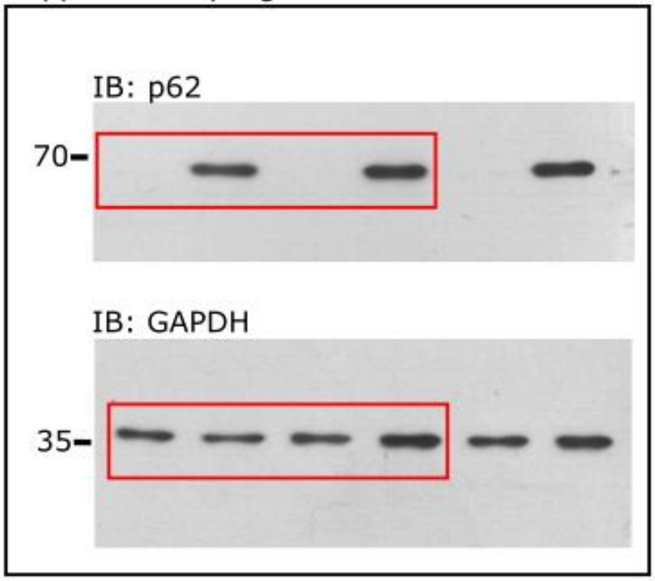

Supplementary Figure 1g

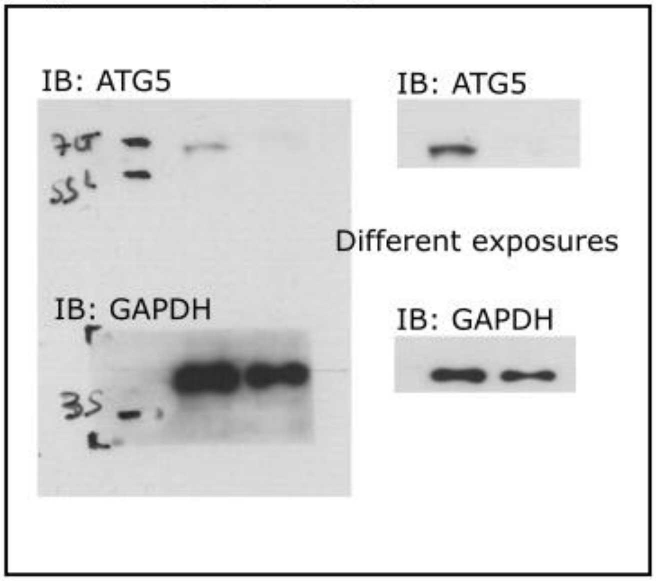

Uncropped immunoblots from Supplementary Figure 1

Supplementary Figure 2h

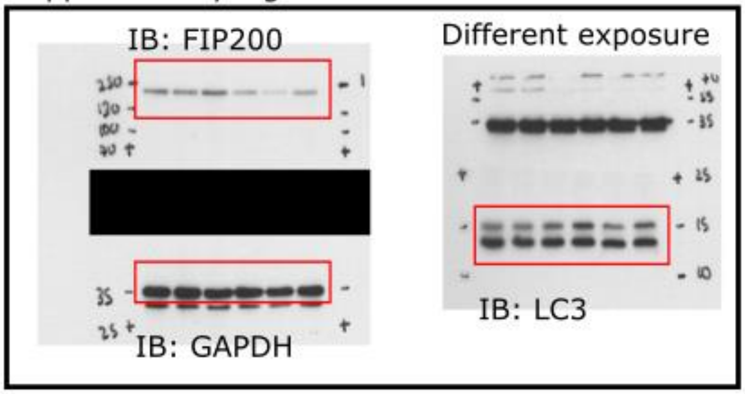

Supplementary Figure 2k

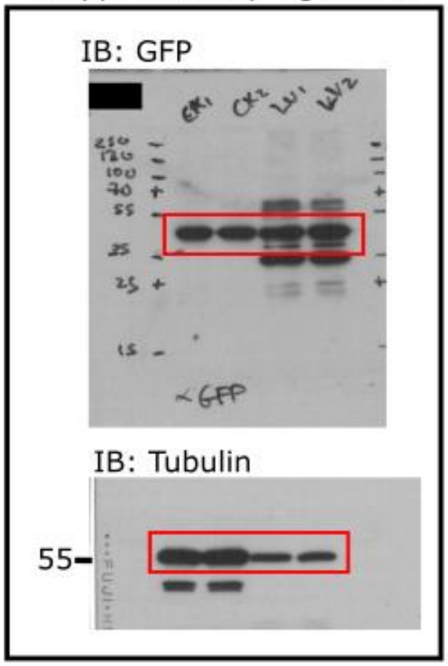

Supplementary Figure 2n

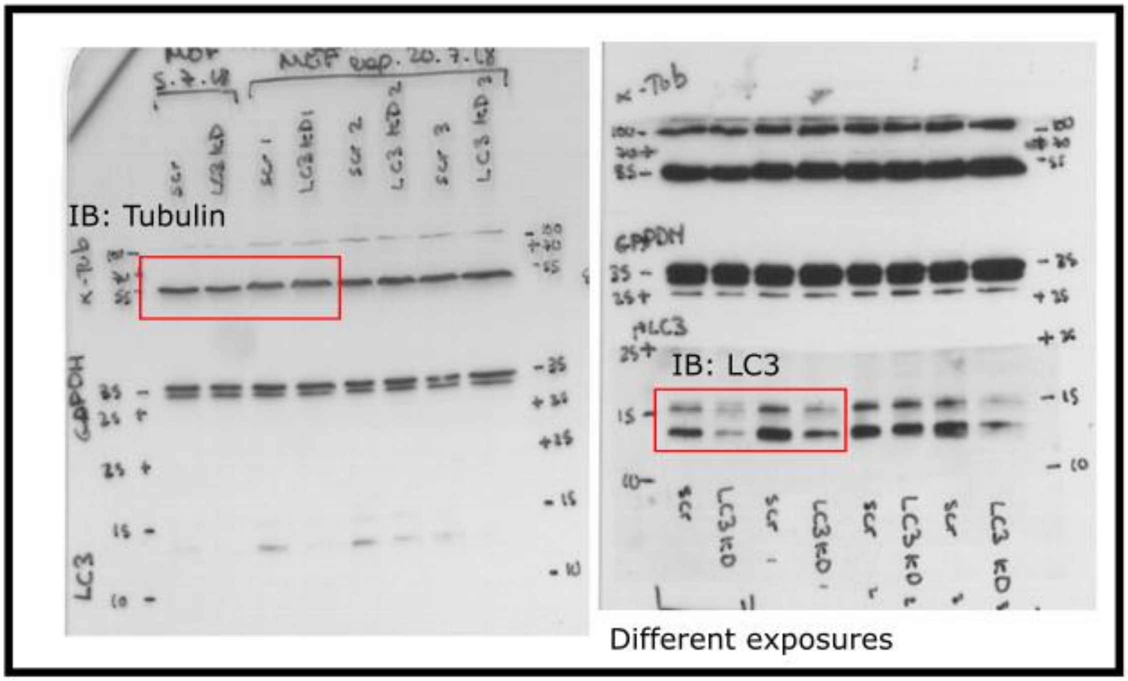

Supplementary Figure 3g

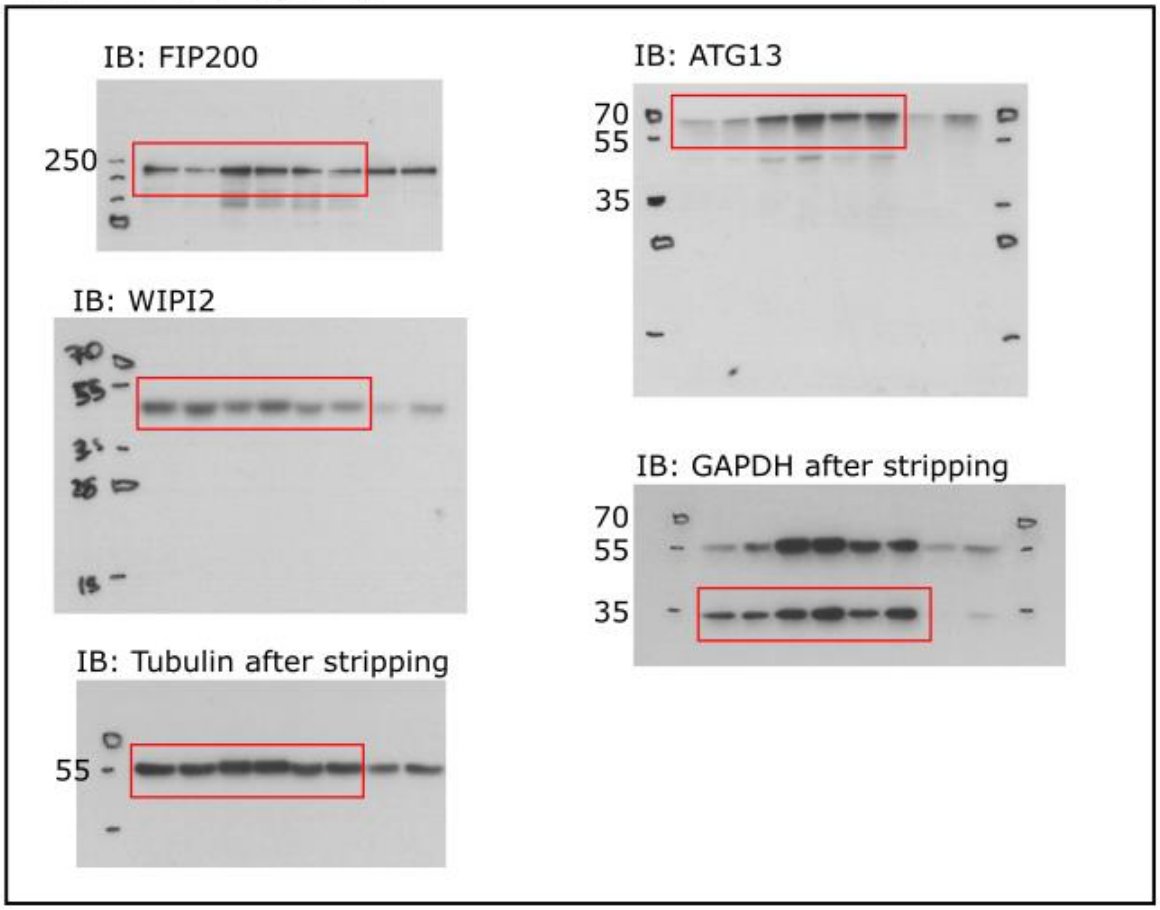

Uncropped immunoblots from Supplementary Figure 3

Supplementary Figure 4a

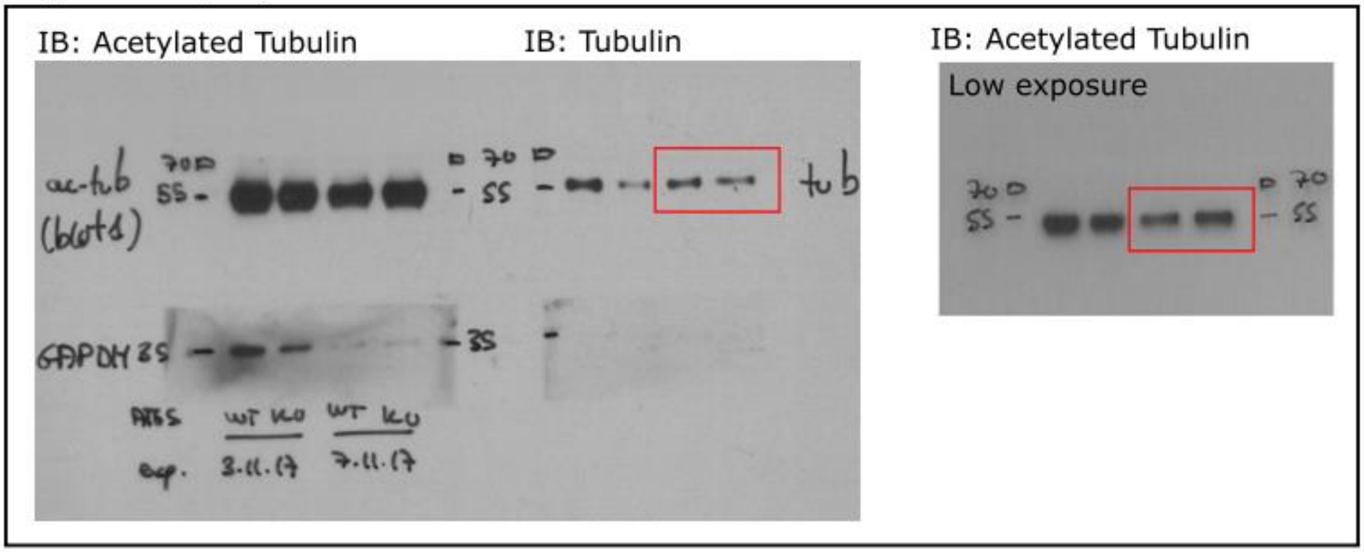

Supplementary Figure 4e

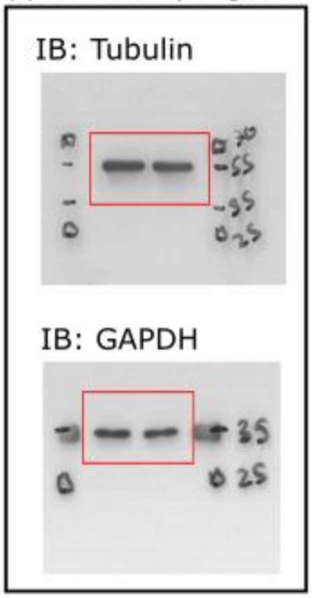

Supplementary Figure 4g

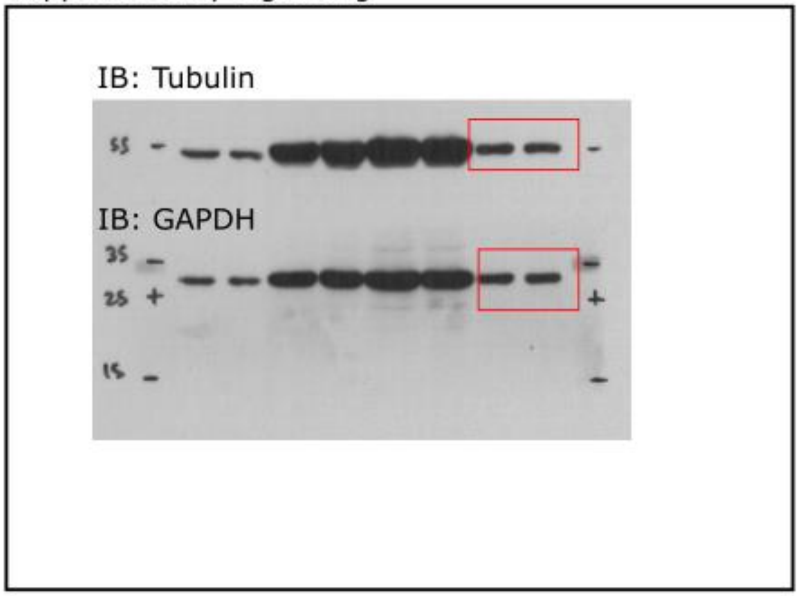

Supplementary Figure 5a

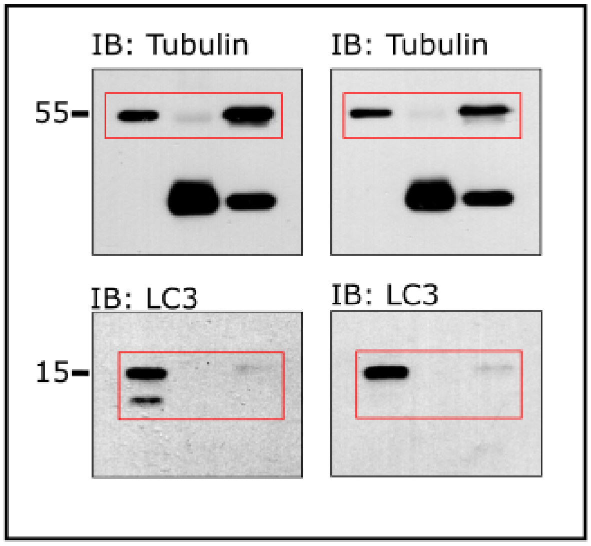

Supplementary Figure 5b

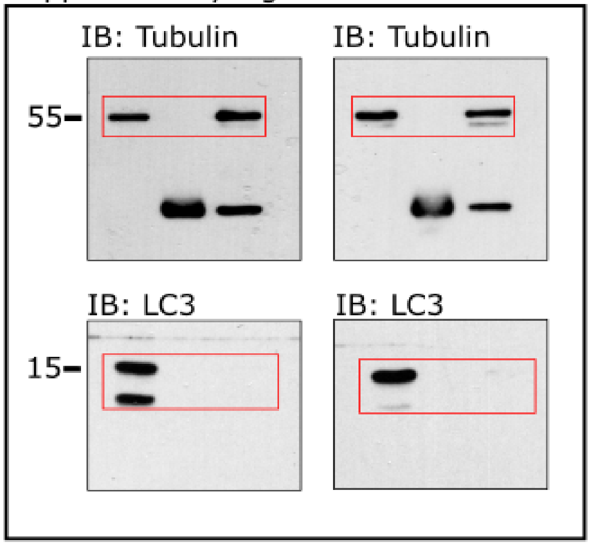

Uncropped immunoblots from Supplementary Figure 5

Supplementary Figure 6f

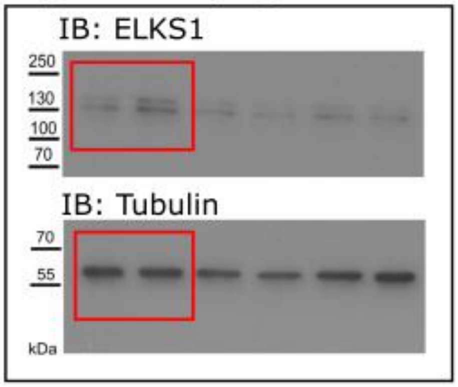

Supplementary Figure 6h

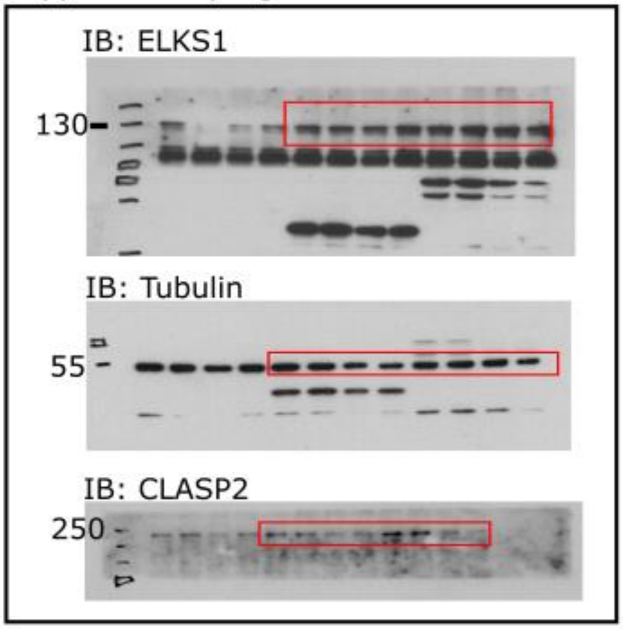

Supplementary Figure 6k

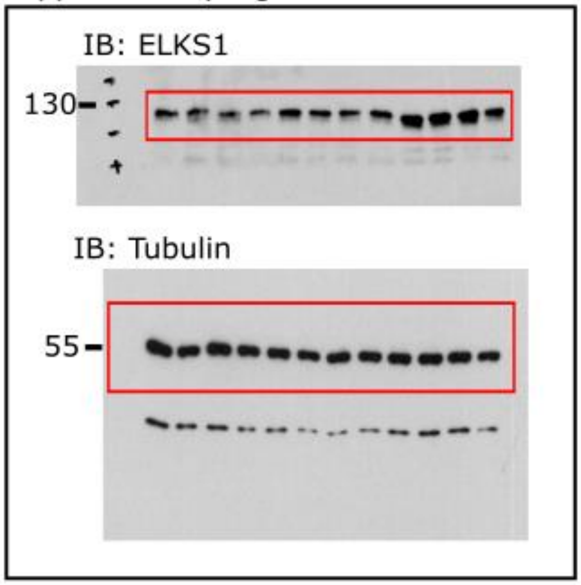

Supplementary Figure 6l

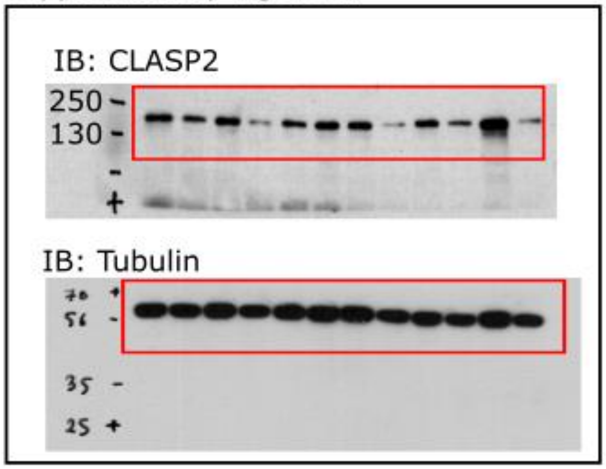

Supplementary Figure 6n

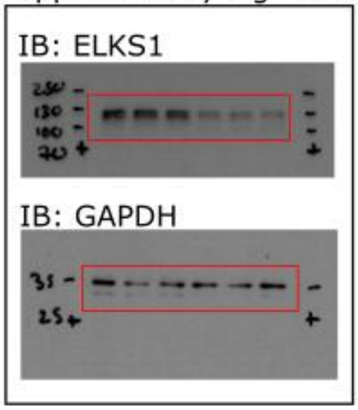

Supplementary Figure 6s

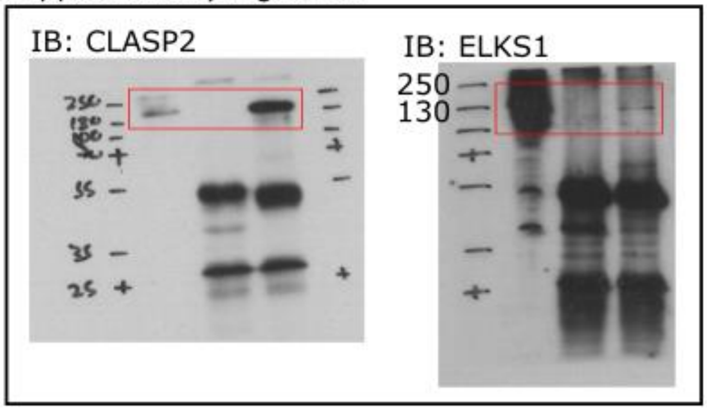

Supplementary Figure 7e

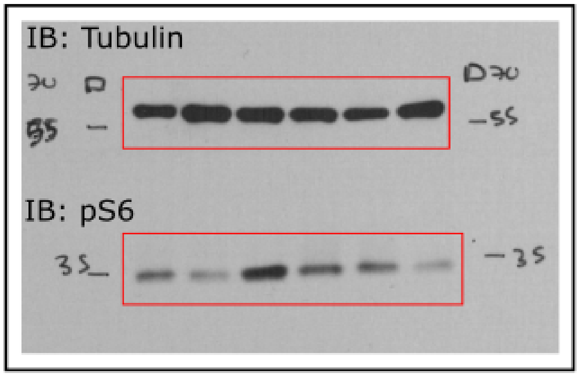

Uncropped immunoblots from Supplementary Figure 7

Supplementary figure 8. Uncropped original scans of WB membranes

Supplementary references:

1. Kye, M.J. *et al.* NMDA mediated contextual conditioning changes miRNA expression. *PloS one* **6**, e24682-e24682 (2011).
2. Kononenko, N.L. *et al.* Compromised fidelity of endocytic synaptic vesicle protein sorting in the absence of stonin 2. *Proceedings of the National Academy of Sciences of the United States of America* **110**, E526-535 (2013).
3. Kononenko, N.L. *et al.* Retrograde transport of TrkB-containing autophagosomes via the adaptor AP-2 mediates neuronal complexity and prevents neurodegeneration. *Nature Communications* **8**, 1-16 (2017).
