# Supplementary material for: Autophagy lipidation machinery regulates axonal microtubule dynamics but is dispensable for survival of mammalian neurons
Source: Nat Commun. 2020 Mar 24;11:1535. doi: 10.1038/s41467-020-15287-9 (PMC7093409; doi:10.1038/s41467-020-15287-9)
Supplement: Supplementary file 3 — Reporting Summary [file 41467_2020_15287_MOESM3_ESM.pdf]

nature research

Corresponding author(s): Natalia Kononenko

Last updated by author(s): Feb 13, 2020

Reporting Summary

Nature Research wishes to improve the reproducibility of the work that we publish. This form provides structure for consistency and transparency in reporting. For further information on Nature Research policies, see Authors & Referees and the Editorial Policy Checklist .

Statistics

For all statistical analyses, confirm that the following items are present in the figure legend, table legend, main text, or Methods section.

n/a	Confirmed	
☐	☒	The exact sample size (*n*) for each experimental group/condition, given as a discrete number and unit of measurement
☐	☒	A statement on whether measurements were taken from distinct samples or whether the same sample was measured repeatedly
☐	☒	The statistical test(s) used AND whether they are one- or two-sided<br>*Only common tests should be described solely by name; describe more complex techniques in the Methods section.*
☒	☐	A description of all covariates tested
☐	☒	A description of any assumptions or corrections, such as tests of normality and adjustment for multiple comparisons
☐	☒	A full description of the statistical parameters including central tendency (e.g. means) or other basic estimates (e.g. regression coefficient) AND variation (e.g. standard deviation) or associated estimates of uncertainty (e.g. confidence intervals)
☐	☒	For null hypothesis testing, the test statistic (e.g. *F*, *t*, *r*) with confidence intervals, effect sizes, degrees of freedom and *P* value noted<br>*Give P values as exact values whenever suitable.*
☒	☐	For Bayesian analysis, information on the choice of priors and Markov chain Monte Carlo settings
☒	☐	For hierarchical and complex designs, identification of the appropriate level for tests and full reporting of outcomes
☒	☐	Estimates of effect sizes (e.g. Cohen's *d*, Pearson's *r*), indicating how they were calculated

Our web collection on statistics for biologists contains articles on many of the points above.

Software and code

Policy information about availability of computer code

Data collection	Leica LAS X Version 2.0.0.14332; Micro-Manager1.4; 7500 Fast Software v2.0.6; Evos Software (EVOS FL Auto2, Life Technologies), EthoVision®XT Noldus, Camera OneView 4K 16 bit (Gatan), DigitalMicrograph (Gatan, JEOL JEM2100PLUS)
Data analysis	FIJI ImageJ 1.52p; KymoMaker (http://www.pharm.hokudai.ac.jp/shinkei/Kymomaker.html); Microsoft Excel 2016; WAVE version 2.6.0. (Seahorse assay); Maxquant version 1.5.3.8 (Mass spectrometry data), GraphPad Pris 8.0.1.

For manuscripts utilizing custom algorithms or software that are central to the research but not yet described in published literature, software must be made available to editors/reviewers. We strongly encourage code deposition in a community repository (e.g. GitHub). See the Nature Research guidelines for submitting code & software for further information.

Data

Policy information about availability of data

All manuscripts must include a data availability statement. This statement should provide the following information, where applicable:

- Accession codes, unique identifiers, or web links for publicly available datasets
- A list of figures that have associated raw data
- A description of any restrictions on data availability

All data supporting the findings described in this study are available from the corresponding author upon reasonable request. Source data from all representative blots shown in this study as well as data underlying all quantitative analysis performed in this study are provided with the manuscript as a Source Data file.
Data generated from mass spectrometry have been deposited in the ProteomeXchange Consortium via the PRIDE (PubMed ID: 26527722) partner repository with the dataset identifier PXD011279.

Field-specific reporting

Please select the one below that is the best fit for your research. If you are not sure, read the appropriate sections before making your selection.

☒ Life sciences ☐ Behavioural & social sciences ☐ Ecological, evolutionary & environmental sciences

For a reference copy of the document with all sections, see nature.com/documents/nr-reporting-summary-flat.pdf

Life sciences study design

All studies must disclose on these points even when the disclosure is negative.

Sample size	Sample sizes were not chosen based on pre-specified effect size. Instead, multiple independent experiments were carried out using several sample replicates as detailed in the figure legends. For all experiments there was enough statistical power to detect the corresponding effect size.
Data exclusions	Data was only excluded when the quality of the sample was not optimal. Predefined quality criteria were: in living samples- vacuolization or other signs of cellular degeneration, normal cell morphology and protein transport (which is usually hampered in unhealthy cells) and image streams that are in focus; proper sample preparation and mounting in fixed samples.
Replication	Each experiment was replicated a minimum of three times and data was reliably reproduced with each replication attempt.
Randomization	Mice were genotyped for allocation into control (Cre negative) or experimental (Cre positive) groups. Within each group, all animals were generally used for experimental procedures, so no randomization was applied.
Blinding	Animals were genotyped prior to experiments, i.e. no blinding was used to allocate experimental groups. Immunofluorescence image data collection and analysis of axons was not blinded since a axonal swellings phenotype differentiated controls from KO groups. Data analysis of indistinguishable cellular structures, such as dendrites and soma of the neuron, were performed blinded. Western blot data were not blindly analysed since either KO protein or siRNA treatment were assessed for each experiment.

Reporting for specific materials, systems and methods

We require information from authors about some types of materials, experimental systems and methods used in many studies. Here, indicate whether each material, system or method listed is relevant to your study. If you are not sure if a list item applies to your research, read the appropriate section before selecting a response.

Materials & experimental systems

n/a	Involved in the study	
☐	☒	Antibodies
☐	☒	Eukaryotic cell lines
☒	☐	Palaeontology
☐	☒	Animals and other organisms
☒	☐	Human research participants
☒	☐	Clinical data

Methods

n/a	Involved in the study	
☒	☐	ChIP-seq
☒	☐	Flow cytometry
☒	☐	MRI-based neuroimaging

Antibodies

Antibodies used

Rabbit polyclonal anti-ATG16L1 MBL Cat# PM040, Lot. 017
Rabbit monoclonal (clone EPR1755(2)) anti-ATG5 abcam Cat# ab108327, Lot. GR312199-7
Rabbit polyclonal anti-ATG9 Novus Biologicals Cat# NB110-56893
Rabbit polyclonal anti-ATG13 Arigo (Biomol GmbH) Cat# ARG55112.050, Lot. 81210
Guinea pig polyclonal anti-Bassoon Synaptic Systems Cat# 141 004, Lot. 1-19
Rabbit polyclonal anti-CASPASE-3 cleaved (Asp175) Cell Signaling Cat# 9661, Lot. 45
Rabbit polyclonal anti-c-FOS Synaptic Systems Cat# 226 003, Lot. 22600313-41
Rabbit polyclonal anti-CLASP2 Millipore Cat# ABT263, Lot. 3040386
Goat polyclonal anti-DNCT1 abcam Cat# ab11806
Rabbit polyclonal anti-ELKS1 Novus Biologicals Cat# NBP1-88178
Rabbit monoclonal anti-FIP200 Cell Signaling Cat# 12436, Lot. 1
Mouse monoclonal anti-GAPDH (clone 71.1) Sigma-Aldrich Cat# G8795
Chicken polyclonal anti-GFP abcam Cat# ab13970, Lot. GR3190550-15
Mouse monoclonal (clone is not provided) anti-GFP Takara Bio Clontech Cat# 632375

Rabbit polyclonal anti-LC3B Novus Biologicals Cat# NB600-1384SS, Lot. CK-1
Mouse monoclonal (clone 4E12) anti-LC3A/B MBL Cat# M152-3, Lot. 051
Mouse monoclonal anti-MAP2 (clone HM-2) Sigma-Aldrich Cat# M9942, Lot. 036M4804V
Mouse monoclonal anti-MAPK activated (cloane MAPK-YT) Sigma-Aldrich Cat# M8159, Lot. 065M4806V
Rabbit polyclonal anti-mCherry abcam Cat# ab167453, Lot. GR3265215-2
Mouse monoclonal anti-mCherry (clone 1C51) Novus Biologicals Cat# NBP1-96752
Rabbit polyclonal anti-P-S6 Ribosomal Protein (Ser235/236) Cell Signaling Cat# 2211, Lot. 23
Guinea pig polyclonal anti-p62 Progen Cat# GP62-C, Lot. 703241-02
Rabbit monoclonal anti-P-AKT (clone D9E) Cell Signaling Cat# 4060, Lot.19
Rabbit monoclonal anti-RAB7 (clone D95F2) Cell Signaling Cat#9367S
Rabbit polyclonal anti-SYB2 Synaptic Systems Cat# 104 202
Mouse monoclonal anti-Synapsin 1 (clone 46.1) Synaptic Systems Cat# 106 001
Rabbit polyclonal anti-P-TRKB (Y816) abcam Cat# ab75173
Mouse monoclonal anti-α-Tubulin (clone 3A2) Synaptic Systems Cat# 302 211, Lot. 1-6
Mouse monoclonal anti- beta-3-Tubulin (clone 2G10-TB3) eBioscience Cat# 14-4510-80, Lot. 4315809
Rabbit monoclonal anti-α-Tubulin acetylated (Lys40, clone D20G3) Cell Signaling Cat# 5335, Lot. 5
Mouse monoclonal anti-α-Tubulin tyrosinated (clone TUB-1A2) Sigma-Aldrich Cat# T9028
Rabbit polyclonal anti-α-Tubulin detyrosinated Millipore Cat# AB3201, Lot: 2886375
Rabbit polyclonal anti-α-Tubulin Δ2 Synaptic Systems Cat# 302 213, Lot: 30221313
Rabbit antiserum anti-Ubiquitin Sigma-Aldrich Cat# U5379
Mouse monoclonal anti-WIPI2 (clone MCA5780GA) BioRad Cat# AB_10845951, Lot. 1801
Goat anti-Mouse IgG (H+L) peroxidase-conjugated Jackson ImmunoResearch Cat# 115-035-003
Goat anti-Mouse IgG, light chain specific, peroxidase conjugated Jackson ImmunoResearch Cat# 115-035-174
Goat anti-Rabbit IgG (H+L) peroxidase-conjugated Jackson ImmunoResearch Cat# 111-035-003
Goat anti-Guinea Pig IgG (H+L) peroxidase-conjugated Jackson ImmunoResearch Cat# 106-035-003, Lot. 124397
Normal Rabbit IgG Cell Signaling Cat# 2729, Lot. 8
Normal Mouse IgG Millipore Cat# 12-371, Lot. 2757162
Alexa Fluor 488 Goat anti-Mouse IgG Thermo Fisher Sci Cat# A-11029; Lot. 1942237
Alexa Fluor 488 Goat anti-Rabbit IgG Thermo Fisher Sci Cat# A-11034, Lot. 1937195
Alexa Fluor 488 Goat anti-Chicken IgG Thermo Fisher Sci Cat# A-11039, Lot. 1691381
Alexa Fluor 568 Goat anti-Mouse IgG Thermo Fisher Sci Cat# A-11031, Lot. 1946341
Alexa Fluor 568 Goat anti-Rabbit IgG Thermo Fisher Sci Cat# A-11036, Lot. 1705911
Alexa Fluor 568 Donkey anti-Goat IgG Thermo Fisher Sci Cat# A-11057, Lot. 2044862
Alexa Fluor 647 Goat anti-Mouse IgG Thermo Fisher Sci Cat# A-21236, Lot. 1705800
Alexa Fluor 647 Goat anti-Rabbit IgG Thermo Fisher Sci Cat# A-21245, Lot. 2098544
Alexa Fluor 647 Goat anti-Guinea Pig IgG Thermo Fisher Sci Cat# A-21450, Lot. 2110845
Alexa Fluor 647 Goat anti-Rat IgG Thermo Fisher Sci Cat# A-21247, Lot. 2005938
Abberior STAR 635P Goat anti-mouse IgG Abberior Cat#ST635P

Validation

Rabbit polyclonal anti-ATG16L1 MBL Cat# PM040: manufacturer validation (https://ruo.mbl.co.jp/bio/dtl/A/?pcd=PM040).
Seto, S., Tsujimura, K., Horii, T., et al. Autophagy adaptor protein p62/SQSTM1 and autophagy-related gene Atg5 mediate autophagosome formation in response to Mycobacterium tuberculosis infection in dendritic cells. PLoS One 8(12) (2013). Tested by WB in mouse cells.

Rabbit monoclonal anti-ATG5 abcam Cat# ab108327: manufacturer validation (https://www.abcam.com/apg5latg5-antibody-epr17552-ab108327.html). Ma, Z., Li, F., Chen, L. et al. Autophagy promotes hepatic differentiation of hepatic progenitor cells by regulating the Wnt/β-catenin signaling pathway. J Mol Hist 50, 75–90 (2019). Tested by WB in mouse cells

Rabbit polyclonal anti-ATG9 Novus Biologicals Cat# NB110-56893: manufacturer validation (https://www.novusbio.com/products/atg9a-antibody_nb110-56893#datasheet). Kaushik, S., Cuervo, A. Degradation of lipid droplet-associated proteins by chaperone-mediated autophagy facilitates lipolysis. Nat Cell Biol 17, 759–770 (2015). Tested by ICC in mouse cells.

Rabbit polyclonal anti-ATG13 Arigo (Biomol GmbH) Cat# ARG55112.050: manufacturer validation (https://www.arigobio.com/anti-ATG13-antibody-ATG13-ARG10995.html). Tested in WB in mouse cells.

Guinea pig polyclonal anti-Bassoon Synaptic Systems Cat# 141 004: manufacturer validation (https://www.sysy.com/products/bassoon/facts-141004.php). Jäpel, M., Gerth, F., Sakaba, T., et al. Intersectin-Mediated Clearance of SNARE Complexes Is Required for Fast Neurotransmission. Cell reports 30 (2), 409-420 (2020). Tested by ICC in mouse cells.

Rabbit polyclonal anti-CASPASE-3 cleaved (Asp175) Cell Signaling Cat# 9661: manufacturer validation (https://www.cellsignal.com/products/primary-antibodies/cleaved-caspase-3-asp175-antibody/9661). Liu, Z., Wang, X., et al. TNFα-induced Up-regulation of Ascl2 Affects the Differentiation and Proliferation of Neural Stem Cells. Aging ans Disease (2019). Tested in IHC in mouse tissue. Lachance, V., Wang, Q., et al. Autophagy protein NRBF2 has reduced expression in Alzheimer's brains and modulates memory and amyloid-beta homeostasis in mice. Mol Neurodegeneration (2019). Tested by WB in brain mouse samples.

Rabbit polyclonal anti-c-FOS Synaptic Systems Cat# 226 003: manufacturer validation (https://www.sysy.com/products/c-fos/facts-226003.php). Grippo RM, et al., Dopamine Signaling in the Suprachiasmatic Nucleus Enables Weight Gain Associated with Hedonic Feeding. Current biology (2019). Tested by IHC in mouse cells.

Rabbit polyclonal anti-CLASP2 Millipore Cat# ABT263: manufacture validation (https://www.sigmaaldrich.com/catalog/product/mm/abt263?lang=es&region=ES). No previously cited. Validated this paper by either knockdown of CLASP2 via siRNA (WB and ICC), and co-localization with GFP-CLASP2 transfected cells (ICC).

Goat polyclonal anti-DNCT1 abcam Cat# ab11806: manufacturer validation (https://www.abcam.com/dctn1p150-glued-antibody-ab11806.html). Magnani E., et al. Interaction of tau protein with the dynactin complex. EMBO J (2007). Tested by ICC/IF in mouse cells.

Rabbit polyclonal anti-ELKS1 Novus Biologicals Cat# NBP1-88178: manufacturer validation (https://www.novusbio.com/products/elks-antibody_nbp1-88178). No previously cited. Validated in the lab by either knockdown of ELKS via siRNA (WB and ICC (this paper)), and co-localization with tdTomato-ELKS1 transfected cells (ICC).

Rabbit monoclonal anti-FIP200 Cell Signaling Cat# 12436: manufacturer validation (https://www.cellsignal.com/products/primary-antibodies/fip200-d10d11-rabbit-mab/12436?Ns=product.displayName%7C0&N=4294959755+4294956287&Nrpp=200&fromPage=plp). Lim J et al., Autophagy regulates inflammatory programmed cell death via turnover of RHIM-domain proteins. eLIFE (2019). Tested by WB in mouse cells.

Mouse monoclonal anti-GAPDH Sigma-Aldrich Cat# G8795: manufacturer validation (https://www.sigmaaldrich.com/catalog/product/sigma/g8795?lang=es&region=ES). Naoya Yuhki et. al Comparative genome organization of human, murine, and feline MHC class II region. Genome research (2003). Tested by WB in mouse cells.

Chicken polyclonal anti-GFP abcam Cat# ab13970: manufacturer validation (https://www.abcam.com/gfp-antibody-ab13970.html). Handara G et al. The role of agrin, Lrp4 and MuSK during dendritic arborization and synaptogenesis in cultured embryonic CNS neurons. Dev Biol (2019). Tested by ICC in mouse cells.

Mouse monoclonal anti-GFP Takara Bio Clontech Cat# 632375: manufacturer validation (https://www.takarabio.com/search-results?term=632375). Previously not cited.

Rabbit polyclonal anti-LC3B Novus Biologicals Cat# NB600-1384SS: manufacturer validation (https://www.novusbio.com/products/lc3b-antibody_nb600-1384). He X., et al. RNF34 functions in immunity and selective mitophagy by targeting MAVS for autophagic degradation. The EMBO Journal (2019). Tested by WB in mouse cells.

Mouse monoclonal anti-LC3A/B MBL Cat# M152-3: manufacturer validation (https://ruo.mbl.co.jp/bio/e/dtl/A/?pcd=M152-3). Kovacs JR et al. Autophagy promotes T-cell survival through degradation of proteins of the cell death machinery. Cell Death Differ. (2012). Tested by ICC in mouse cells.

Mouse monoclonal anti-MAP2 Sigma-Aldrich Cat# M9942: manufacturer validation (https://www.sigmaaldrich.com/catalog/product/sigma/m9942?lang=es&region=ES). Chen YL., et al. Modulation of mGluR-dependent MAP1B translation and AMPA receptor endocytosis by microRNA miR-146a-5p.The Journal of Neuroscience 33(21), 9013-9020, (2013). Tested by ICC in mouse cells.

Mouse monoclonal anti-MAPK activated (P-ERK-1&2) Sigma-Aldrich Cat# M8159: manufacturer validation (https://www.sigmaaldrich.com/catalog/product/sigma/m8159?lang=es&region=ES). Blizinsky KD et al., Reversal of dendritic phenotypes in 16p11. 2 microduplication mouse model neurons by pharmacological targeting of a network hub.Proceedings of the National Academy of Sciences of the USA (2016). Tested by WB in mouse cells.

Rabbit polyclonal anti-mCherry abcam Cat# ab167453: manufacturer validation (https://www.abcam.com/mcherry-antibody-ab167453.html). Hoffmann S., et al. Light-Activated ROS Production Induces Synaptic Autophagy. J Neurosci (2019). Tested by ICC and WB in mouse cells.

Mouse monoclonal anti-mCherry Novus Biologicals Cat# NBP1-96752: manufacturer validation (https://www.novusbio.com/products/mcherry-antibody-1c51_nbp1-96752). Choi, E et al., Mitotic Regulators and the SHP2-MAPK Pathway Promote Insulin Receptor Endocytosis and Feedback Regulation of Insulin Signaling. Nat Commun (2018). Tested by ICC/IF in mouse cells.

Rabbit polyclonal anti-P-S6 Ribosomal Protein (Ser235/236) Cell Signaling Cat# 2211: manufacturer validation (https://www.cellsignal.com/products/primary-antibodies/phospho-s6-ribosomal-protein-ser235-236-antibody/2211). Tripathi, P., Rodríguez-Muela, N., et al., Reactive Astrocytes Promote ALS-like Degeneration and Intracellular Protein Aggregation in Human Motor Neurons by Disrupting Autophagy through TGF-β1.S tem Cell Reports (2017). Tested by WB in mouse cells.

Guinea pig polyclonal anti-p62 Progen Cat# GP62-C: manufacturer validation (https://www.progen.com/anti-p62-sqstm1-c-terminus-guinea-pig-polyclonal-serum.html). Domke LM and Franke WW. The cell–cell junctions of mammalian testes: II. The lamellar smooth muscle monolayer cells of the peritubular wall are laterally connected by vertical adherens junctions—a novel architectonic cell–cell junction system. Cell and Tissue Research (2019). Tested by WB and ICC/IF in mouse cells.

Rabbit monoclonal anti-P-AKT (Ser473) Cell Signaling Cat# 4060: manufacturer validation (https://www.cellsignal.com/products/primary-antibodies/phospho-akt-ser473-d9e-xp-rabbit-mab/4060). Tian, S., Jia, W., et al. Dual-specificity tyrosine

phosphorylation-regulated kinase 1A ameliorates insulin resistance in neurons by up-regulating IRS-1 expression.The Journal of Biological Chemistry (2019). Tested by WB in mouse cells.

Rabbit monoclonal anti-RAB7 Cell Signaling Cat#9367S: manufacturer validation (https://www.cellsignal.com/products/primary-antibodies/rab7-d95f2-xp-rabbit-mab/9367). Hosaka, K., Yang, Y., et al..Dual roles of endothelial FGF-2-FGFR1-PDGF-BB and perivascular FGF-2-FGFR2-PDGFRβ signaling pathways in tumor vascular remodeling. Cell Discovery (2018). Tested by ICC in mouse cells.

Rabbit polyclonal anti-SYB2 Synaptic Systems Cat# 104 202: manufacturer validation (https://www.sysy.com/products/s-brevin2/facts-104202.php). Liu C, Kershberg L, Wang J, Schneeberger S, Kaeser PS. Dopamine Secretion Is Mediated by Sparse Active Zone-like Release Sites. Cell (2018). Tested by WB and ICC in mouse cells.

Mouse monoclonal anti-Synapsin 1 Synaptic Systems Cat# 106 001: manufacturer validation (https://www.sysy.com/products/synapsin/facts-106001.php). Brockhaus J, Brüggen B, Missler M. Imaging and Analysis of Presynaptic Calcium Influx in Cultured Neurons Using synGCaMP6f. Frontiers in synaptic neuroscience (2019). Tested by ICC in mouse cells.

Rabbit polyclonal anti-P-TRKB (Y816) abcam Cat# ab75173: manufacturer validation (https://www.citeab.com/antibodies/756719-ab75173-anti-trkb-phospho-y816-antibody). Li, W., Calfa, G., et al. Activity-dependent BDNF release and TRPC signaling is impaired in hippocampal neurons of Mecp2 mutant mice. Proceedings of the National Academy of Sciences of the United States of America (2012). Tested by IF, IHC and WB in mouse cells.

Mouse monoclonal anti-α-Tubulin Synaptic Systems Cat# 302 211: manufacturer validation (https://www.sysy.com/products/tubulin/facts-302211.php). Götzke H et al., The ALFA-tag is a highly versatile tool for nanobody-based bioscience applications. Nature communications (2019). Tested by WB in mouse.

Mouse monoclonal anti- beta-3-Tubulin Thermo Fisher Sci Cat# 14-4510-80: manufacturer validation (https://www.thermofisher.com/antibody/product/beta-3-Tubulin-Antibody-clone-2G10-TB3-Monoclonal/14-4510-82).

Rabbit monoclonal anti-α-Tubulin acetylated (Lys40) Cell Signaling Cat# 5335: manufacturer validation (https://www.cellsignal.com/products/primary-antibodies/acetyl-a-tubulin-lys40-d20g3-xp-rabbit-mab/5335). Sheu, J. R., Hsieh, C. Y., et al.. HDAC6 dysfunction contributes to impaired maturation of adult neurogenesis in vivo: vital role on functional recovery after ischemic stroke. Journal of Biomedical Science (2019). Tested by WB in mouse cells.

Mouse monoclonal anti-α-Tubulin tyrosinated Sigma-Aldrich Cat# T9028: manufacturer validation (https://www.sigmaaldrich.com/catalog/product/sigma/t9028?lang=es&region=ES). Cho Y., et al. Filamin A is required in injured axons for HDAC5 activity and axon regeneration. J Biol Chem (2015). Tested by WB in mouse cells.

Rabbit polyclonal anti-α-Tubulin detyrosinated Millipore Cat# AB3201: manufacturer validation (https://www.merckmillipore.com/ES/es/product/Anti-Tubulin-Antibody-Detyrosinated,MM_NF-AB3201?ReferrerURL=https%3A%2F%2Fwww.google.com%2F). Herms, A., et al. AMPK activation promotes lipid droplet dispersion on detyrosinated microtubules to increase mitochondrial fatty acid oxidation. Nat comm (2015). Tested by IF. Schock, EN., et al. Using the avian mutant talpid2 as a disease model for understanding the oral-facial phenotypes of oral-facial-digital syndrome. Disease models & mechanisms (2015). Tested by WB in mouse.

Rabbit polyclonal anti-α-Tubulin Δ2 Synaptic Systems Cat# 302 213: manufacturer validation (https://www.sysy.com/products/tubulin/facts-302213.php).

Rabbit antiserum anti-Ubiquitin Sigma-Aldrich Cat# U5379: manufacturer validation (https://www.sigmaaldrich.com/catalog/product/sigma/u5379?lang=es&region=ES). Sewduth, RN., et al. The ubiquitin ligase PDZRN3 is required for vascular morphogenesis through Wnt/planar cell polarity signalling. Nat Comm (2014). Tested by WB in mouse samples. Ayaki T., et al. Immunoreactivity of valosin-containing protein in sporadic amyotrophic lateral sclerosis and in a case of its novel mutant. Acta Neuropathol Commun. (2014). Tested by IHC.

Mouse monoclonal anti-WIPI2 BioRad Cat# AB_10845951: manufacturer validation (https://www.bio-rad-antibodies.com/monoclonal/human-wipi2-antibody-2a2-mca5780.html?f=purified). Hannah EJ., et al. Mammalian Atg18 (WIPI2) localizes to omegasome-anchored phagophores and positively regulates LC3 lipidation. Autophagy (2010). Tested by WB and ICC in mouse cells.

Goat anti-Mouse IgG (H+L) peroxidase-conjugated Jackson ImmunoResearch Cat# 115-035-003: manufacturer validation (https://www.jacksonimmuno.com/catalog/products/115-035-003). Du, X., Zhang, Z., Zheng, X. et al. An electrochemical biosensor for the detection of epithelial-mesenchymal transition. Nat Commun 11, 192 (2020).

Goat anti-Mouse IgG, light chain specific, peroxidase conjugated Jackson ImmunoResearch Cat# 115-035-174: manufacturer validation (https://www.jacksonimmuno.com/catalog/products/115-035-174). : Sutton, L.P., Muntean, B.S., Ostrovskaya, O., Zucca, S., Dao, M., Orlandi, C., et al. (2019) NF1-cAMP signaling dissociates cell type–specific contributions of striatal medium spiny neurons to reward valuation and motor control. PLoS Biol 17 (10): e3000477.

Goat anti-Rabbit IgG (H+L) peroxidase-conjugated Jackson ImmunoResearch Cat# 111-035-003: manufacturer validation (https://www.jacksonimmuno.com/catalog/products/111-035-003). Eisenberg-Lerner, A., Benyair, R., Hizkiahou, N., Nudel, N., Maor, R., Kramer, M. P., … Merbl, Y. Golgi organization is regulated by proteasomal degradation. Nat comm, 11(1), 409. (2020)

Goat anti-Guinea Pig IgG (H+L) peroxidase-conjugated Jackson ImmunoResearch Cat# 106-035-003: manufacturer validation (https://www.jacksonimmuno.com/catalog/products/106-035-003). Saito, T., Kuma, A., Sugiura, Y. et al. Autophagy regulates lipid metabolism through selective turnover of NCoR1. Nat Commun 10, 1567 (2019)

Normal Rabbit IgG Cell Signaling Cat# 2729: manufacturer validation (https://www.cellsignal.com/products/primary-antibodies/normal-rabbit-igg/2729). Lückel, C., Picard, F., Raifer, H. et al. IL-17+ CD8+ T cell suppression by dimethyl fumarate associates with clinical response in multiple sclerosis. Nat Commun 10, 5722 (2019).

Normal Mouse IgG Millipore Cat# 12-371: manufacturer validation (https://www.merckmillipore.com/ES/es/product/Normal-Mouse-IgG,MM_NF-12-371?ReferrerURL=https%3A%2F%2Fwww.google.com%2F). amayo, A., Duong, H., Robles, M. et al. Histone monoubiquitination by Clock–Bmal1 complex marks Per1 and Per2 genes for circadian feedback. Nat Struct Mol Biol 22, 759–766 (2015).

Alexa Fluor 488 Goat anti-Mouse IgG Thermo Fisher Sci Cat# A-11029: manufacturer validation (https://www.thermofisher.com/antibody/product/Goat-anti-Mouse-IgG-H-L-Highly-Cross-Adsorbed-Secondary-Antibody-Polyclonal/A-11029). Shumilov, A., Tsai, M., Schlosser, Y. et al. Epstein–Barr virus particles induce centrosome amplification and chromosomal instability. Nat Commun 8, 14257 (2017).

Alexa Fluor 488 Goat anti-Rabbit IgG Thermo Fisher Sci Cat# A-11034: manufacturer validation (https://www.thermofisher.com/antibody/product/Goat-anti-Rabbit-IgG-H-L-Highly-Cross-Adsorbed-Secondary-Antibody-Polyclonal/A-11034). Lopez, J., Lim, R., Cruceanu, C. et al. miR-1202 is a primate-specific and brain-enriched microRNA involved in major depression and antidepressant treatment. Nat Med 20, 764–768 (2014).

Alexa Fluor 488 Goat anti-Chicken IgG Thermo Fisher Sci Cat# A-11039: manufacturer validation (https:/7www.thermofisher.com/antibody/product/Goat-anti-Chicken-IgY-H-L-Secondary-Antibody-Polyclonal/A-11039). Quadrato, G., Nguyen, T., Macosko, E. et al. Cell diversity and network dynamics in photosensitive human brain organoids. Nature 545, 48–53 (2017).

Alexa Fluor 568 Goat anti-Mouse IgG Thermo Fisher Sci Cat# A-11031: manufacturer validation (https://www.thermofisher.com/antibody/product/Goat-anti-Mouse-IgG-H-L-Highly-Cross-Adsorbed-Secondary-Antibody-Polyclonal/A-11031). van de Ven, R., de Groot, J., Park, D. et al. p120-catenin prevents multinucleation through control of MKLP1-dependent RhoA activity during cytokinesis. Nat Commun 7, 13874 (2016).

Alexa Fluor 568 Goat anti-Rabbit IgG Thermo Fisher Sci Cat# A-11036: manufacturer validation (https://www.thermofisher.com/antibody/product/Goat-anti-Rabbit-IgG-H-L-Highly-Cross-Adsorbed-Secondary-Antibody-Polyclonal/A-11036). Hoch, N., Hanzlikova, H., Rulten, S. et al. XRCC1 mutation is associated with PARP1 hyperactivation and cerebellar ataxia. Nature 541, 87–91 (2017).

Alexa Fluor 568 Donkey anti-Goat IgG Thermo Fisher Sci Cat# A-11057: manufacturer validation (https://www.thermofisher.com/antibody/product/Donkey-anti-Goat-IgG-H-L-Cross-Adsorbed-Secondary-Antibody-Polyclonal/A-11057). Eggenschwiler, R., Moslem, M., Fráguas, M. et al. Improved bi-allelic modification of a transcriptionally silent locus in patient-derived iPSC by Cas9 nickase. Sci Rep 6, 38198 (2016).

Alexa Fluor 647 Goat anti-Mouse IgG Thermo Fisher Sci Cat# A-21236: manufacturer validation (https://www.thermofisher.com/antibody/product/Goat-anti-Mouse-IgG-H-L-Highly-Cross-Adsorbed-Secondary-Antibody-Polyclonal/A-21236). Kim, S., Im, S., Oh, S. et al. Anisotropically organized three-dimensional culture platform for reconstruction of a hippocampal neural network. Nat Commun 8, 14346 (2017).

Alexa Fluor 647 Goat anti-Rabbit IgG Thermo Fisher Sci Cat# A-21245: manufacturer validation (https://www.thermofisher.com/antibody/product/Goat-anti-Rabbit-IgG-H-L-Highly-Cross-Adsorbed-Secondary-Antibody-Polyclonal/A-21245). Loo, L., Bougen-Zhukov, N. & Tan, W. Early spatiotemporal-specific changes in intermediate signals are predictive of cytotoxic sensitivity to TNFα and co-treatments. Sci Rep 7, 43541 (2017).

Alexa Fluor 647 Goat anti-Guinea Pig IgG Thermo Fisher Sci Cat# A-21450: manufacturer validation (https://www.thermofisher.com/antibody/product/Goat-anti-Guinea-Pig-IgG-H-L-Highly-Cross-Adsorbed-Secondary-Antibody-Polyclonal/A-21450). Bramini, M., Sacchetti, S., Armirotti, A., et al. Graphene Oxide Nanosheets Disrupt Lipid Composition, Ca^{2+} Homeostasis, and Synaptic Transmission in Primary Cortical Neurons. ACS Nano 10 (7), 7154-7171 (2016).

Alexa Fluor 647 Goat anti-Rat IgG Thermo Fisher Sci Cat# A-21247: manufacturer validation (https://www.thermofisher.com/antibody/product/Goat-anti-Rat-IgG-H-L-Cross-Adsorbed-Secondary-Antibody-Polyclonal/A-21247). Quadrato, G., Nguyen, T., Macosko, E. et al. Cell diversity and network dynamics in photosensitive human brain organoids. Nature 545, 48–53 (2017)

Abberior STAR 635P Goat anti-mouse IgG Abberior Cat#ST635P: manufacturer validation (https://www.abberior.com/jtl-shop/Abberior-STAR-635P). Mazkereth, N., Rocca, F., Schubert, J.R., et al. Immunobiology 221 (12), 1395-1406 (2016).

Eukaryotic cell lines

Policy information about cell lines

Cell line source(s)	HEK293T were obtained from Prof. M. Lammers (CECAD, Cologne) and were originally from ATCC, NSC34 cells (CLU140, Cedarlane) were obtained from Prof. B.Wirth (University Hospital, Cologne), MEF cells were obtained form Prof. T. Langer (MPI for Biology of Ageing, Cologne)
Authentication	Cell lines were authenticated by morphology
Mycoplasma contamination	Cell lines were not tested for mycoplasma contamination
Commonly misidentified lines (See ICLAC register)	No commonly misidentified cell lines were used in the study.

Animals and other organisms

Policy information about studies involving animals; ARRIVE guidelines recommended for reporting animal research

Laboratory animals	Mus Musculus, C57Bl/6, both sexes, postnatal days P0-P5, 12-13 weeks old
Wild animals	This study did not involve wild animals
Field-collected samples	This study did not involve samples collected from the field
Ethics oversight	All animal experiments were reviewed and approved by the ethics committee of the "Landesamtes für Natur, Umwelt- und Verbraucherschutz des Landes Nordrhein-Westfalen", Cologne (2015.A578, 2016.A041, 2016.A451)

Note that full information on the approval of the study protocol must also be provided in the manuscript.
